# Nonpharmaceutical Measures for Pandemic Influenza in Nonhealthcare Settings—Personal Protective and Environmental Measures

**DOI:** 10.3201/eid2605.190994

**Published:** 2020-05

**Authors:** Jingyi Xiao, Eunice Y. C. Shiu, Huizhi Gao, Jessica Y. Wong, Min W. Fong, Sukhyun Ryu, Benjamin J. Cowling

**Affiliations:** University of Hong Kong, Hong Kong, China

**Keywords:** influenza, pandemic influenza, influenza virus, viruses, respiratory infections, nonpharmaceutical measures, nonhealthcare settings, personal protective measures, environmental measures, meta-analysis, public health

## Abstract

There were 3 influenza pandemics in the 20th century, and there has been 1 so far in the 21st century. Local, national, and international health authorities regularly update their plans for mitigating the next influenza pandemic in light of the latest available evidence on the effectiveness of various control measures in reducing transmission. Here, we review the evidence base on the effectiveness of nonpharmaceutical personal protective measures and environmental hygiene measures in nonhealthcare settings and discuss their potential inclusion in pandemic plans. Although mechanistic studies support the potential effect of hand hygiene or face masks, evidence from 14 randomized controlled trials of these measures did not support a substantial effect on transmission of laboratory-confirmed influenza. We similarly found limited evidence on the effectiveness of improved hygiene and environmental cleaning. We identified several major knowledge gaps requiring further research, most fundamentally an improved characterization of the modes of person-to-person transmission.

Influenza pandemics occur at irregular intervals when new strains of influenza A virus spread in humans (*1*). Influenza pandemics cause considerable health and social impact that exceeds that of typical seasonal (interpandemic) influenza epidemics. One of the characteristics of influenza pandemics is the high incidence of infections in all age groups because of the lack of population immunity. Although influenza vaccines are the cornerstone of seasonal influenza control, specific vaccines for a novel pandemic strain are not expected to be available for the first 5–6 months of the next pandemic. Antiviral drugs will be available in some locations to treat more severe infections but are unlikely to be available in the quantities that might be required to control transmission in the general community. Thus, efforts to control the next pandemic will rely largely on nonpharmaceutical interventions.

Most influenza virus infections cause mild and self-limiting disease; only a small fraction of case-patients require hospitalization. Therefore, influenza virus infections spread mainly in the community. Influenza virus is believed to be transmitted predominantly by respiratory droplets, but the size distribution of particles responsible for transmission remains unclear, and in particular, there is a lack of consensus on the role of fine particle aerosols in transmission (*2*,*3*). In healthcare settings, droplet precautions are recommended in addition to standard precautions for healthcare personnel when interacting with influenza patients and for all visitors during influenza seasons (*4*). Outside healthcare settings, hand hygiene is recommended in most national pandemic plans (*5*), and medical face masks were a common sight during the influenza pandemic in 2009. Hand hygiene has been proven to prevent many infectious diseases and might be considered a major component in influenza pandemic plans, whether or not it has proven effectiveness against influenza virus transmission, specifically because of its potential to reduce other infections and thereby reduce pressure on healthcare services.

In this article, we review the evidence base for personal protective measures and environmental hygiene measures, and specifically the evidence for the effectiveness of these measures in reducing transmission of laboratory-confirmed influenza in the community. We also discuss the implications of the evidence base for inclusion of these measures in pandemic plans.

## Methods and Results

We conducted systematic reviews to evaluate the effectiveness of personal protective measures on influenza virus transmission, including hand hygiene, respiratory etiquette, and face masks, and a systematic review of surface and object cleaning as an environmental measure (Table 1). We searched 4 databases (Medline, PubMed, EMBASE, and CENTRAL) for literature in all languages. We aimed to identify randomized controlled trials (RCTs) of each measure for laboratory-confirmed influenza outcomes for each of the measures because RCTs provide the highest quality of evidence. For respiratory etiquette and surface and object cleaning, because of a lack of RCTs for laboratory-confirmed influenza, we also searched for RCTs reporting effects of these interventions on influenza-like illness (ILI) and respiratory illness outcomes and then for observational studies on laboratory-confirmed influenza, ILI, and respiratory illness outcomes. For each review, 2 authors (E.Y.C.S. and J.X.) screened titles and abstracts and reviewed full texts independently.

**Table 1:** Summary of literature searches for systematic review on personal and environmental nonpharmaceutical interventions for pandemic influenza*

Types of interventions	No. studies identified	Study designs included†	Main findings
Hand hygiene	11	RCT	The evidence from RCTs suggested that hand hygiene interventions do not have a substantial effect on influenza transmission.
Respiratory etiquette	0	NA	We did not identify research evaluating the effectiveness of respiratory etiquette on influenza transmission.
Face masks	10	RCT	The evidence from RCTs suggested that the use of face masks either by infected persons or by uninfected persons does not have a substantial effect on influenza transmission.
Surface and object cleaning	3	RCT, observational studies	There was a limited amount of evidence suggesting that surface and object cleaning does not have a substantial effect on influenza transmission.

*NA, not available; RCT randomized controlled trial. †In these systematic reviews, we prioritized RCTs, and only considered observational studies if there were a small number of RCTs. Our rationale was that with evidence from a larger number of RCTs, additional evidence from observational studies would be unlikely to change overall conclusions.

We performed meta-analysis for hand hygiene and face mask interventions and estimated the effect of these measures on laboratory-confirmed influenza prevention by risk ratios (RRs). We used a fixed-effects model to estimate the overall effect in a pooled analysis or subgroup analysis. No overall effect would be generated if there was considerable heterogeneity on the basis of *I*^2^ statistic >75% (*6*). We performed quality assessment of evidence on hand hygiene and face mask interventions by using the GRADE (Grading of Recommendations Assessment, Development and Evaluation) approach (*7*). We provide additional details of the search strategies, selection of articles, summaries of the selected articles, and quality assessment (Appendix).

### Personal Protective Measures

#### Hand Hygiene

**Figure 1:**
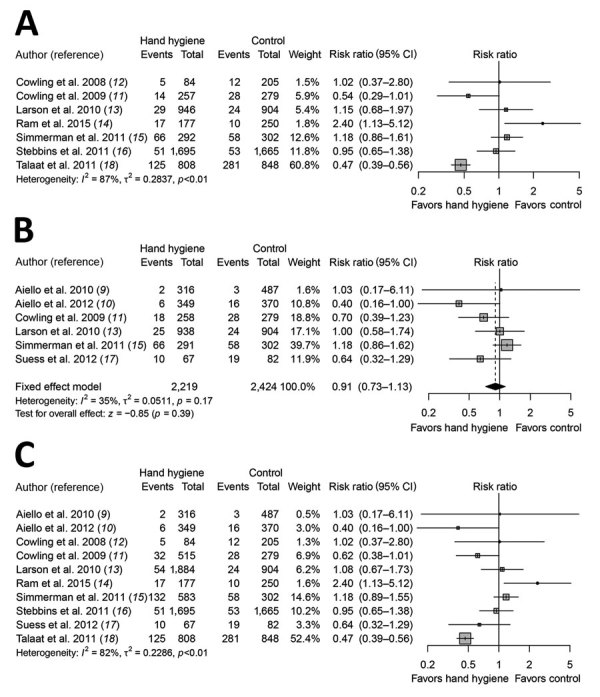
Meta-analysis of risk ratios for the effect of hand hygiene with or without face mask use on laboratory-confirmed influenza from 10 randomized controlled trials with >11,000 participants. A) Hand hygiene alone; B) hand hygiene and face mask; C) hand hygiene with or without face mask. Pooled estimates were not made if there was high heterogeneity (*I*^2^ >75%). Squares indicate risk ratio for each of the included studies, horizontal lines indicate 95% CIs, dashed vertical line indicates pooled estimation of risk ratio, and diamond indicates pooled estimation of risk ratio. Diamond width corresponds to the 95% CI.

We identified a recent systematic review by Wong et al. on RCTs designed to assess the efficacy of hand hygiene interventions against transmission of laboratory-confirmed influenza (*8*). We used this review as a starting point and then searched for additional literature published after 2013; we found 3 additional eligible articles published during the search period of January 1, 2013–August 13, 2018. In total, we identified 12 articles (*9*–*20*), of which 3 articles were from the updated search and 9 articles from Wong et al. (*8*). Two articles relied on the same underlying dataset (*16*,*19*); therefore, we counted these 2 articles as 1 study, which resulted in 11 RCTs. We further selected 10 studies with >10,000 participants for inclusion in the meta-analysis (Figure 1). We excluded 1 study from the meta-analysis because it provided estimates of infection risks only at the household level, not the individual level (*20*). We did not generate an overall pooled effect of hand hygiene only or of hand hygiene with or without face mask because of high heterogeneity in individual estimates (*I*^2^ 87 and 82%, respectively). The effect of hand hygiene combined with face masks on laboratory-confirmed influenza was not statistically significant (RR 0.91, 95% CI 0.73–1.13; *I*^2^ = 35%, p = 0.39). Some studies reported being underpowered because of limited sample size, and low adherence to hand hygiene interventions was observed in some studies.

We further analyzed the effect of hand hygiene by setting because transmission routes might vary in different settings. We found 6 studies in household settings examining the effect of hand hygiene with or without face masks, but the overall pooled effect was not statistically significant (RR 1.05, 95% CI 0.86–1.27; *I*^2^ = 57%, p = 0.65) (Appendix Figure 4) (*11*–*15*,*17*). The findings of 2 studies in school settings were different (Appendix Figure 5). A study conducted in the United States (*16*) showed no major effect of hand hygiene, whereas a study in Egypt (*18*) reported that hand hygiene reduced the risk for influenza by >50%. A pooled analysis of 2 studies in university residential halls reported a marginally significant protective effect of a combination of hand hygiene plus face masks worn by all residents (RR 0.48, 95% CI 0.21–1.08; *I*^2^ = 0%, p = 0.08) (Appendix Figure 6) (*9*,*10*).

In support of hand hygiene as an effective measure, experimental studies have reported that influenza virus could survive on human hands for a short time and could transmit between hands and contaminated surfaces (*2*,*21*). Some field studies reported that influenza A(H1N1)pdm09 and influenza A(H3N2) virus RNA and viable influenza virus could be detected on the hands of persons with laboratory-confirmed influenza (*22**,**23*), supporting the potential of direct and indirect contact transmission to play a role in the spread of influenza. Other experimental studies also demonstrated that hand hygiene could reduce or remove infectious influenza virus from human hands (*24**,**25*). However, results from our meta-analysis on RCTs did not provide evidence to support a protective effect of hand hygiene against transmission of laboratory-confirmed influenza. One study did report a major effect, but in this trial of hand hygiene in schools in Egypt, running water had to be installed and soap and hand-drying material had to be introduced into the intervention schools as part of the project (*18*). Therefore, the impact of hand hygiene might also be a reflection of the introduction of soap and running water into primary schools in a lower-income setting. If one considers all of the evidence from RCTs together, it is useful to note that some studies might have underestimated the true effect of hand hygiene because of the complexity of implementing these intervention studies. For instance, the control group would not typically have zero knowledge or use of hand hygiene, and the intervention group might not adhere to optimal hand hygiene practices (*11*,*13*,*15*).

Hand hygiene is also effective in preventing other infectious diseases, including diarrheal diseases and some respiratory diseases (*8**,**26*). The need for hand hygiene in disease prevention is well recognized among most communities. Hand hygiene has been accepted as a personal protective measure in >50% of national preparedness plans for pandemic influenza (*5*). Hand hygiene practice is commonly performed with soap and water, alcohol-based hand rub, or other waterless hand disinfectants, all of which are easily accessible, available, affordable, and well accepted in most communities. However, resource limitations in some areas are a concern when clean running water or alcohol-based hand rub are not available. There are few adverse effects of hand hygiene except for skin irritation caused by some hand hygiene products (*27*). However, because of certain social or religious practices, alcohol-based hand sanitizers might not be permitted in some locations (*28*). Compliance with proper hand hygiene practice tends to be low because habitual behaviors are difficult to change (*29*). Therefore, hand hygiene promotion programs are needed to advocate and encourage proper and effective hand hygiene.

#### Respiratory Etiquette

Respiratory etiquette is defined as covering the nose and mouth with a tissue or a mask (but not a hand) when coughing or sneezing, followed by proper disposal of used tissues, and proper hand hygiene after contact with respiratory secretions (*30*). Other descriptions of this measure have included turning the head and covering the mouth when coughing and coughing or sneezing into a sleeve or elbow, rather than a hand. The rationale for not coughing into hands is to prevent subsequent contamination of other surfaces or objects (*31*). We conducted a search on November 6, 2018, and identified literature that was available in the databases during 1946–November 5, 2018. We did not identify any published research on the effectiveness of respiratory etiquette in reducing the risk for laboratory-confirmed influenza or ILI. One observational study reported a similar incidence rate of self-reported respiratory illness (defined by >1 symptoms: cough, congestion, sore throat, sneezing, or breathing problems) among US pilgrims with or without practicing respiratory etiquette during the Hajj (*32*). The authors did not specify the type of respiratory etiquette used by participants in the study. A laboratory-based study reported that common respiratory etiquette, including covering the mouth by hands, tissue, or sleeve/arm, was fairly ineffective in blocking the release and dispersion of droplets into the surrounding environment on the basis of measurement of emitted droplets with a laser diffraction system (*31*).

Respiratory etiquette is often listed as a preventive measure for respiratory infections. However, there is a lack of scientific evidence to support this measure. Whether respiratory etiquette is an effective nonpharmaceutical intervention in preventing influenza virus transmission remains questionable, and worthy of further research.

#### Face Masks

**Figure 2:**
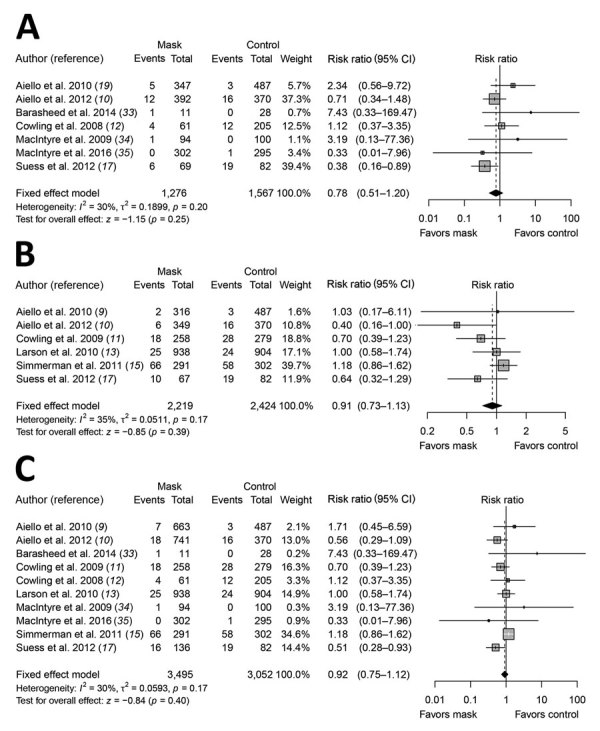
Meta-analysis of risk ratios for the effect of face mask use with or without enhanced hand hygiene on laboratory-confirmed influenza from 10 randomized controlled trials with >6,500 participants. A) Face mask use alone; B) face mask and hand hygiene; C) face mask with or without hand hygiene. Pooled estimates were not made if there was high heterogeneity (*I*^2^ >75%). Squares indicate risk ratio for each of the included studies, horizontal lines indicate 95% CIs, dashed vertical lines indicate pooled estimation of risk ratio, and diamonds indicate pooled estimation of risk ratio. Diamond width corresponds to the 95% CI.

In our systematic review, we identified 10 RCTs that reported estimates of the effectiveness of face masks in reducing laboratory-confirmed influenza virus infections in the community from literature published during 1946–July 27, 2018. In pooled analysis, we found no significant reduction in influenza transmission with the use of face masks (RR 0.78, 95% CI 0.51–1.20; *I*^2^ = 30%, p = 0.25) (Figure 2). One study evaluated the use of masks among pilgrims from Australia during the Hajj pilgrimage and reported no major difference in the risk for laboratory-confirmed influenza virus infection in the control or mask group (*33*). Two studies in university settings assessed the effectiveness of face masks for primary protection by monitoring the incidence of laboratory-confirmed influenza among student hall residents for 5 months (*9*,*10*). The overall reduction in ILI or laboratory-confirmed influenza cases in the face mask group was not significant in either studies (*9*,*10*). Study designs in the 7 household studies were slightly different: 1 study provided face masks and P2 respirators for household contacts only (*34*), another study evaluated face mask use as a source control for infected persons only (*35*), and the remaining studies provided masks for the infected persons as well as their close contacts (*11*–*13*,*15*,*17*). None of the household studies reported a significant reduction in secondary laboratory-confirmed influenza virus infections in the face mask group (*11*–*13*,*15*,*17*,*34*,*35*). Most studies were underpowered because of limited sample size, and some studies also reported suboptimal adherence in the face mask group.

Disposable medical masks (also known as surgical masks) are loose-fitting devices that were designed to be worn by medical personnel to protect accidental contamination of patient wounds, and to protect the wearer against splashes or sprays of bodily fluids (*36*). There is limited evidence for their effectiveness in preventing influenza virus transmission either when worn by the infected person for source control or when worn by uninfected persons to reduce exposure. Our systematic review found no significant effect of face masks on transmission of laboratory-confirmed influenza.

We did not consider the use of respirators in the community. Respirators are tight-fitting masks that can protect the wearer from fine particles (*37*) and should provide better protection against influenza virus exposures when properly worn because of higher filtration efficiency. However, respirators, such as N95 and P2 masks, work best when they are fit-tested, and these masks will be in limited supply during the next pandemic. These specialist devices should be reserved for use in healthcare settings or in special subpopulations such as immunocompromised persons in the community, first responders, and those performing other critical community functions, as supplies permit.

In lower-income settings, it is more likely that reusable cloth masks will be used rather than disposable medical masks because of cost and availability (*38*). There are still few uncertainties in the practice of face mask use, such as who should wear the mask and how long it should be used for. In theory, transmission should be reduced the most if both infected members and other contacts wear masks, but compliance in uninfected close contacts could be a problem (*12*,*34*). Proper use of face masks is essential because improper use might increase the risk for transmission (*39*). Thus, education on the proper use and disposal of used face masks, including hand hygiene, is also needed.

### Environmental Measures

#### Surface and Object Cleaning

For the search period from 1946 through October 14, 2018, we identified 2 RCTs and 1 observational study about surface and object cleaning measures for inclusion in our systematic review (*40*–*42*). One RCT conducted in day care nurseries found that biweekly cleaning and disinfection of toys and linen reduced the detection of multiple viruses, including adenovirus, rhinovirus, and respiratory syncytial virus in the environment, but this intervention was not significant in reducing detection of influenza virus, and it had no major protective effect on acute respiratory illness (*41*). Another RCT found that hand hygiene with hand sanitizer together with surface disinfection reduced absenteeism related to gastrointestinal illness in elementary schools, but there was no major reduction in absenteeism related to respiratory illness (*42*). A cross-sectional study found that passive contact with bleach was associated with a major increase in self-reported influenza (*40*).

Given that influenza virus can survive on some surfaces for prolonged periods (*43*), and that cleaning or disinfection procedures can effectively reduce or inactivate influenza virus from surfaces and objects in experimental studies (*44*), there is a theoretical basis to believe that environmental cleaning could reduce influenza transmission. As an illustration of this proposal, a modeling study estimated that cleaning of extensively touched surfaces could reduce influenza A infection by 2% (*45*). However, most studies of influenza virus in the environment are based on detection of virus RNA by PCR, and few studies reported detection of viable virus.

Although we found no evidence that surface and object cleaning could reduce influenza transmission, this measure does have an established impact on prevention of other infectious diseases (*42*). It should be feasible to implement this measure in most settings, subject to the availability of water and cleaning products. Although irritation caused by cleaning products is limited, safety remains a concern because some cleaning products can be toxic or cause allergies (*40*).

## Discussion

In this review, we did not find evidence to support a protective effect of personal protective measures or environmental measures in reducing influenza transmission. Although these measures have mechanistic support based on our knowledge of how influenza is transmitted from person to person, randomized trials of hand hygiene and face masks have not demonstrated protection against laboratory-confirmed influenza, with 1 exception (*18*). We identified only 2 RCTs on environmental cleaning and no RCTs on cough etiquette.

Hand hygiene is a widely used intervention and has been shown to effectively reduce the transmission of gastrointestinal infections and respiratory infections (*26*). However, in our systematic review, updating the findings of Wong et al. (*8*), we did not find evidence of a major effect of hand hygiene on laboratory-confirmed influenza virus transmission (Figure 1). Nevertheless, hand hygiene might be included in influenza pandemic plans as part of general hygiene and infection prevention.

We did not find evidence that surgical-type face masks are effective in reducing laboratory-confirmed influenza transmission, either when worn by infected persons (source control) or by persons in the general community to reduce their susceptibility (Figure 2). However, as with hand hygiene, face masks might be able to reduce the transmission of other infections and therefore have value in an influenza pandemic when healthcare resources are stretched.

It is essential to note that the mechanisms of person-to-person transmission in the community have not been fully determined. Controversy remains over the role of transmission through fine-particle aerosols (*3*,*46*). Transmission by indirect contact requires transfer of viable virus from respiratory mucosa onto hands and other surfaces, survival on those surfaces, and successful inoculation into the respiratory mucosa of another person. All of these components of the transmission route have not been studied extensively. The impact of environmental factors, such as temperature and humidity, on influenza transmission is also uncertain (*47*). These uncertainties over basic transmission modes and mechanisms hinder the optimization of control measures.

In this review, we focused on 3 personal protective measures and 1 environmental measure. Other potential environmental measures include humidification in dry environments (*48*), increasing ventilation (*49*), and use of upper-room UV light (*50*), but there is limited evidence to support these measures. Further investigations on the effectiveness of respiratory etiquette and surface cleaning through conducting RCTs would be helpful to provide evidence with higher quality; evaluation of the effectiveness of these measures targeting specific population groups, such as immunocompromised persons, would also be beneficial (Table 2). Future cost-effectiveness evaluations could provide more support for the potential use of these measures. Further research on transmission modes and alternative interventions to reduce influenza transmission would be valuable in improving pandemic preparedness. Finally, although our review focused on nonpharmaceutical measures to be taken during influenza pandemics, the findings could also apply to severe seasonal influenza epidemics. Evidence from RCTs of hand hygiene or face masks did not support a substantial effect on transmission of laboratory-confirmed influenza, and limited evidence was available on other environmental measures.

**Table 2:** Knowledge gaps for personal protective and environmental nonpharmaceutical interventions for pandemic influenza*

Intervention	Knowledge gaps	Suggested studies
Hand hygiene	There are major gaps in our knowledge of the mechanisms of person-to-person transmission of influenza, including the role of direct and indirect contact, the degree of viral contamination on hands and various types of surfaces in different settings, and the potential for contact transmission to occur in different locations and under different environmental conditions. There is little information on whether greater reductions in transmission could be possible with combinations of personal intervention (e.g., isolation away from family members as much as possible, plus using face masks and enhancing hand hygiene).	Additional high-quality RCTs of efficacy of hand hygiene against laboratory-confirmed influenza in other nonhealthcare settings, except households and university residential halls, would be valuable. In particular, studies in school settings are needed to solve the discrepancy between the two studies from the United States and Egypt.
Respiratory etiquette	There is no evidence about the quantitative effectiveness of respiratory etiquette against influenza virus.	RCTs of interventions to demonstrate the effectiveness of respiratory etiquette in reducing influenza transmission would be valuable.
Face mask	There are major gaps in our knowledge of the mechanisms of person-to-person transmission of influenza, including the importance of transmission through droplets of different sizes including small particle aerosols, and the potential for droplet and aerosol transmission to occur in different locations and with environmental conditions.	Additional high-quality RCTs of efficacy of face masks against laboratory-confirmed influenza would be valuable. Effectiveness of face masks or respirator use to prevent influenza prevention in special subpopulation, such as immunocompromised persons, would be valuable.
Surface and object cleaning	The effectiveness of different cleaning products in preventing influenza transmission–in terms of cleaning frequency, cleaning dosage, cleaning time point, and cleaning targeted surface and object material–remains unknown.	RCTs of interventions to demonstrate the effectiveness of surface and object cleaning in reducing influenza transmission would be valuable. Studies that can demonstrate the reduction of environmental detection of influenza virus through cleaning of surfaces and objects would also be valuable.

*RCT, randomized control trial.

Appendix Additional information on nonpharmaceutical measures for pandemic influenza in nonhealthcare settings—personal protective measures and environmental measures.
