# Supplementary material for: Nonpharmaceutical Measures for Pandemic Influenza in Nonhealthcare Settings—Personal Protective and Environmental Measures
Source: Emerg Infect Dis. 2020 May;26(5):967–75. doi: 10.3201/eid2605.190994 (PMC7181938; doi:10.3201/eid2605.190994)
Supplement: Appendix — Additional information on nonpharmaceutical measures for pandemic influenza in nonhealthcare settings—personal protective measures and environmental measures. [file 19-0994-Techapp-s1.pdf]

Article DOI: https://doi.org/10.3201/eid2605.190994

Nonpharmaceutical Measures for Pandemic Influenza in Nonheathcare Settings—Personal Protective and Environmental Measures

Appendix

Hand Hygiene

Terminology

Relevant terminology relating to hand hygiene are shown as follows (Appendix Table 1):

Appendix Table 1. Definition of terms relevant to hand hygiene

Term	Definition
Hand hygiene practices	
Hand hygiene	"A general term referring to any action of hand cleansing" (e.g., handwashing, antiseptic handwash, antiseptic hand rub and surgical hand antisepsis) (*1,2*)
Hand cleansing	"Action of performing hand hygiene for the purpose of physically or mechanically removing dirt, organic material, and/or microorganisms" (*1*)
Handwashing	"Washing hands with plain or antimicrobial soap and water" (*1*)
Antiseptic handwashing	"Washing hands with soap and water, or other detergents containing an antiseptic agent" (*1*)
Antiseptic handrubbing (or handrubbing)	Applying an antiseptic handrub to all surfaces of the hands "to reduce or inhibit the growth of microorganisms without the need for an exogenous source of water and requiring no rinsing or drying with towels or other devices" (*1,2*)
Hand disinfection	"Hand disinfection is extensively used as a term in some parts of the world and can refer to antiseptic handwash, antiseptic handrubbing, hand antisepsis/decontamination/degerming, handwashing with an antimicrobial soap and water, hygienic hand antisepsis, or hygienic handrub" (*1*)
Hand hygiene products	
Alcohol-based (hand) rub (or hand sanitizer)	"An alcohol-containing preparation (liquid, gel or foam) designed for application to the hands to inactivate microorganisms and/or temporarily suppress their growth. Such preparations may contain one or more types of alcohol, other active ingredients with excipients, and humectants" (*1)*
Antimicrobial (medicated) soap	"Soap (detergent) containing an antiseptic agent at a concentration sufficient to inactivate microorganisms and/or temporarily suppress their growth. The detergent activity of such soaps may also dislodge transient microorganisms or other contaminants from the skin to facilitate their subsequent removal by water" (*1*)
Antiseptic hand wipe	"A piece of fabric or paper pre-wetted with an antiseptic used for wiping hands to inactivate and/or remove microbial contamination. They may be considered as an alternative to washing hands with non-antimicrobial soap and water but, because they are not as effective at reducing bacterial counts on HCWs' hands as alcohol-based handrubs or washing hands with an antimicrobial soap and water, they are not a substitute for using an alcohol-based handrub or antimicrobial soap" (*1*)
Plain soap	"Plain soap refers to detergents that do not contain antimicrobial agents or contain low concentrations of antimicrobial agents that are effective solely as preservatives" (*2*)
Other	
Visibly soiled hands	"Hands showing visible dirt or visibly contaminated with proteinaceous material, blood, or other body fluids (e.g., fecal material or urine)" (2)

Search Strategy

We conducted a review of systematic review on 1 August 2018 using the following search terms (Appendix Table 2) to identify literatures that were available from 1946 through July 31, 2018. Four databases (PubMed, Medline, EMBASE, and CENTRAL) were searched. Review selection criteria are systematic reviews published within 5 years studying the effect of hand hygiene interventions on prevention of laboratory-confirmed influenza in community settings. We reviewed literatures of all languages.

Appendix Table 2. Search strategy for hand hygiene

Search terms	Search date	Reviewers
#1: "hand hygiene" OR "hand washing" OR "handwashing" OR "hand-washing" OR "hand-wash" OR "hand wash" OR "handwash" OR "hand sanitize" OR "hand sanitizers" OR "hand sanitizer" OR "hand rub" OR "handrub" OR "hand rubbing" OR "hand cleansing" OR "hand cleans" OR "hand cleanser" OR "hand disinfectant" OR "hand disinfectants" OR "hand disinfection" OR "hand soap" OR "hand wipe"	August 1, 2018 for review of systematic review and August 14, 2018 for additional search	J.X.<br>E.S.
#2: "influenza" OR "flu"		
#3: #1 AND #2		

After identifying the most recent published systematic review, we conducted an additional literature search to capture articles of all languages that were not included in the review. The search was conducted on 14 August 2018 using the same search terms in 4 databases to identify literatures that were available during January 1, 2013–August 13, 2018. Study selection criteria are randomized control trials (RCTs) comparing the effect of hand hygiene interventions with that of no intervention in preventing laboratory-confirmed influenza infections in community settings. Study participants or clusters of participants were assigned prospectively into intervention and control groups using random allocation. A community setting was defined as an open setting without confinement or special care for patients. Articles describing any hand hygiene related interventions were included. Two reviewers (J.X. and E.S.) reviewed retrieved titles and subsequent relevant abstracts independently. Titles and abstract selected by any of the reviewers were included for subsequent screening. Both reviewers reviewed full-text and extracted data for selected studies independently. If a consensus was not reached, further discussion was held or opinion was obtained from a third reviewer.

With a substantial number of randomized controlled trials conducted on hand hygiene, we did not extend the search to observational studies, but we did note the findings from earlier systematic reviews of observational studies of hand hygiene (*4*–*6*).

Risk ratios (RRs) and their 95% confidence intervals (95% CIs) were calculated to estimate the effect of hand hygiene intervention on prevention of laboratory-confirmed influenza.

Heterogeneity of each pooled and subgroup analysis was assessed by I^2 statistics. The overall effect of each pooled and subgroup analysis was estimated by fixed-effect model. If the heterogeneity was high ($I^2 \geq 75\%$), we did not estimate an overall pooled effect.

Findings

Appendix Table 3. Basic characteristics of included studies

Characteristic	No. of studies (%)
Country	
Industrialized	7 (64)
Developing	4 (36)
Setting	
Household	7 (64)
Elementary school	2 (18)
University residential hall	2 (18)
Transmission mode	
Primary	5 (45)
Secondary	6 (55)
Intervention evaluated*	
Hand sanitizer and education	3 (20)
Hand sanitizer, soap and education	1 (7)
Hand sanitizer and face mask	3 (20)
Hand sanitizer, face mask and education	2 (13)
Soap	3 (20)
Soap and education	1 (7)
Soap and face mask	2 (13)
Outcome assessed	
Laboratory-confirmed influenza	11 (100)

*More than one intervention for some studies.

We identified 225 reviews through the search, of which 172 reviews were removed during title and abstract screening. We selected 7 reviews for our analysis after screening the full text. Reasons for exclusion included: reviews considered not systematic, reviews published outside the 5-year time frame, articles in reviews were not RCTs, and the reviews did not evaluate hand hygiene as a study intervention or laboratory-confirmed influenza infection as a study outcome. Among the 7 included reviews, we identified 9 relevant RCT studies, of which all 9 studies were included in a most recently published systematic review and metaanalysis conducted by Wong et al. (*7*). Therefore, we used this review as the reference base of our review of systematic review to evaluate the effect of hand hygiene in reducing the risk for laboratory-confirmed influenza virus infection. The flowchart is shown in Appendix Figure 1.

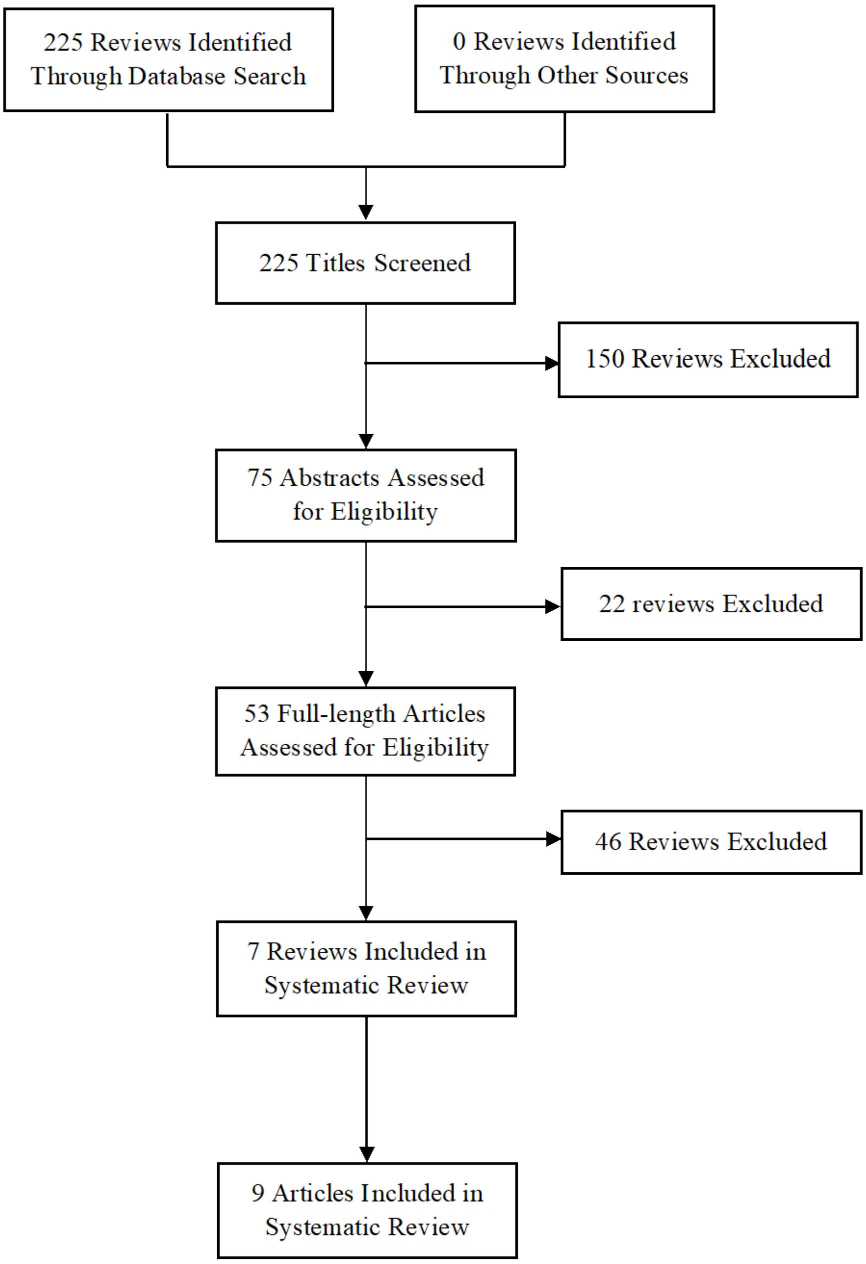

Appendix Figure 1. Flowchart of literature search and article selection for systematic reviews of the effectiveness of hand hygiene against laboratory-confirmed influenza.

Furthermore, we conducted an update search to capture literatures that were not included in the review by Wong et al. (*7*). We identified 352 articles from January 1, 2013 to August 13, 2018. We subsequently removed 319 articles during the title and abstract screening. Reasons for exclusion included: studies were not conducted in community settings, study design was not RCT and studies did not evaluate hand hygiene as a study intervention or laboratory-confirmed influenza infection as a study outcome. We identified 3 articles in this updated search, hence we included a total of 12 articles in our systematic review. Since two articles used the same dataset

to evaluate different research questions (*8*,*9*), we considered these articles as 1 study in our review. Moreover, one article only included secondary infection data in household level but not individual level (*9*), therefore we did not include this in the metaanalysis. To sum up, we included 12 articles in our systematic review and 11 articles in the metaanalysis. The flowchart is shown in Appendix Figure 2.

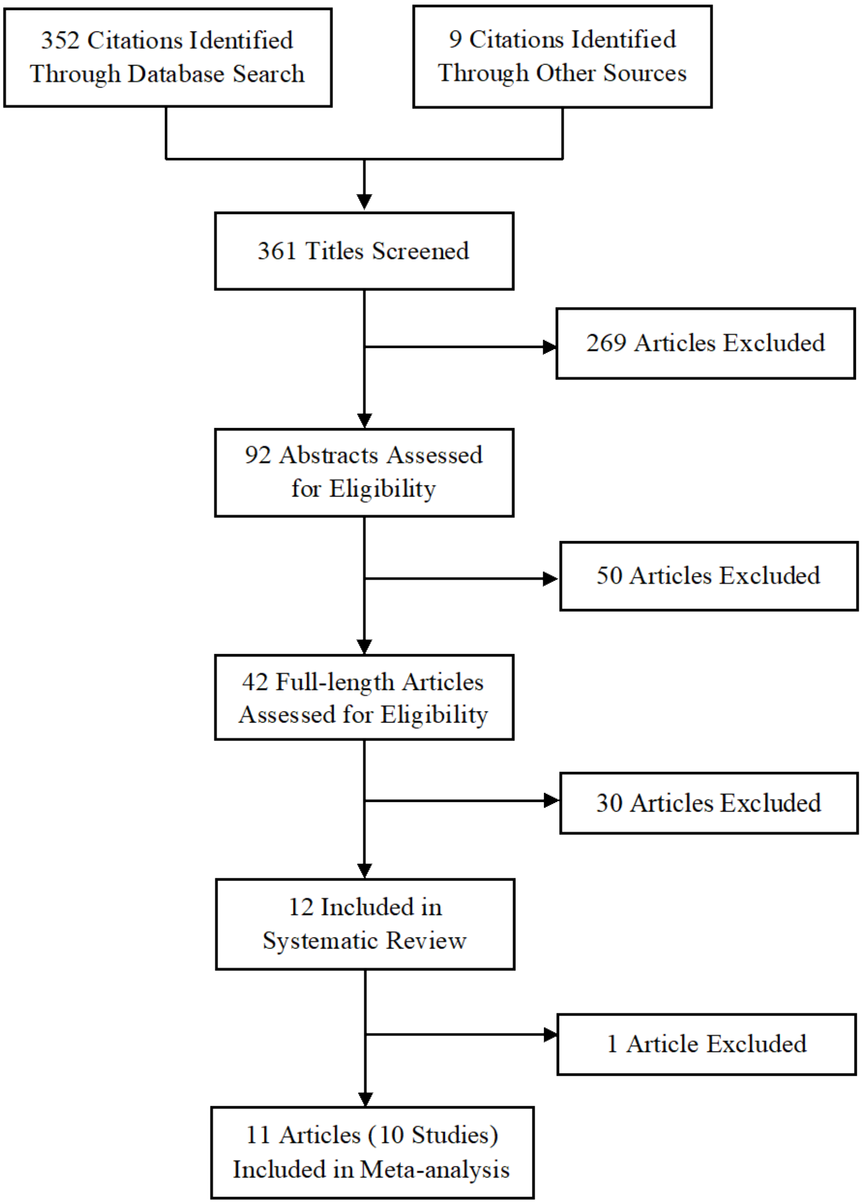

Appendix Figure 2. Flowchart of literature search and study selection for trials of the effectiveness of hand hygiene against laboratory-confirmed influenza.

Among the 11 studies included in the metaanalysis, 7 studies were in household settings (*10*–*16*), 2 studies in elementary school setting (*9*,*17*), and 2 studies in university residential hall

setting (*18,19*). Basic characteristics of the included studies are shown in Appendix Table 3, and detailed study description are shown in Appendix Table 4.

In the pooled analysis, hand hygiene with face mask (risk ratio [RR] 0.91, 95% CI 0.73–1.13; p = 0.39, I^2 = 35%) did not have a significant protective effect in community settings (Appendix Figure 3) (*11,13,15,16,18,19*). Some published studies noted that poor adherence to hand hygiene might lead to underestimation of the true effect of the intervention (*11,13,15*).

Because the relative importance of transmission modes of influenza might vary in different settings, we conducted subgroup analysis based on various settings. In household setting (*11–16*), the efficacy of hand hygiene with or without face mask was not significant (RR 1.05, 95% CI 0.86–1.27; p = 0.65, I^2 = 57%) (Appendix Figure 4). Although the pooled analysis did not identify an overall significant effect of hand hygiene, some household transmission studies reported that early implementation of hand hygiene in the index case after symptom onset might be more effective in preventing secondary infection in the household (*11,16*).

In school settings (*9,17*), total effect was not generated because of high heterogeneity (Appendix Figure 5). In a study in the United States (*9*), the effect of hand hygiene was not significant based on the point estimate of the RR close to 1, whereas a large trial in Egypt reported a reduction of >50% of influenza cases in the intervention group (*17*).

In university residential hall settings (*18,19*), hand hygiene with face mask intervention contributed to 52% RR reduction (RR = 0.48, 95% CI 0.21–1.08; p = 0.08, marginally significant, I^2 = 0%) of laboratory-confirmed influenza infection (Appendix Figure 6).

The results of quality assessment of evidence on hand hygiene intervention using GRADE (Grading of Recommendations Assessment, Development, and Evaluation) approach are shown in Appendix Table 5.

Hand hygiene only

Author	Hand hygiene Events	Hand hygiene Total	Control Events	Control Total	Weight	Risk Ratio	95% C.I.
Cowling et al. 2008	5	84	12	205	1.5%	1.02	[0.37; 2.80]
Cowling et al. 2009	14	257	28	279	5.9%	0.54	[0.29; 1.01]
Larson et al. 2010	29	946	24	904	5.4%	1.15	[0.68; 1.97]
Ram et al. 2015	17	177	10	250	1.8%	2.40	[1.13; 5.12]
Simmerman et al. 2011	66	292	58	302	12.6%	1.18	[0.86; 1.61]
Stebbins et al. 2011	51	1695	53	1665	11.8%	0.95	[0.65; 1.38]
Talaat et al. 2011	125	808	281	848	60.8%	0.47	[0.39; 0.56]

Heterogeneity: $I^2 = 87\%$, $\tau^2 = 0.2837$, $p < 0.01$

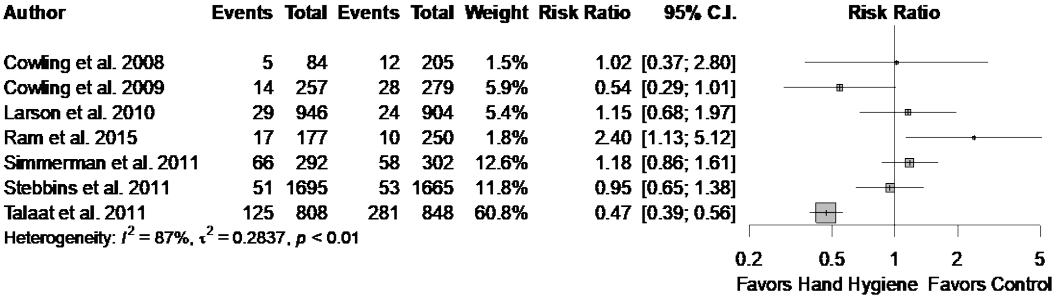

Hand hygiene and facemask

Author	Hand hygiene Events	Hand hygiene Total	Control Events	Control Total	Weight	Risk Ratio	95% C.I.
Aiello et al. 2010	2	316	3	487	1.6%	1.03	[0.17; 6.11]
Aiello et al. 2012	6	349	16	370	10.8%	0.40	[0.16; 1.00]
Cowling et al. 2009	18	258	28	279	18.8%	0.70	[0.39; 1.23]
Larson et al. 2010	25	938	24	904	17.1%	1.00	[0.58; 1.74]
Simmerman et al. 2011	66	291	58	302	39.7%	1.18	[0.86; 1.62]
Suess et al. 2012	10	67	19	82	11.9%	0.64	[0.32; 1.29]
**Fixed effect model**		**2219**		**2424**	**100.0%**	**0.91**	**[0.73; 1.13]**

Heterogeneity: $I^2 = 35\%$, $\tau^2 = 0.0511$, $p = 0.17$
Test for overall effect: $z = -0.85$ ($p = 0.39$)

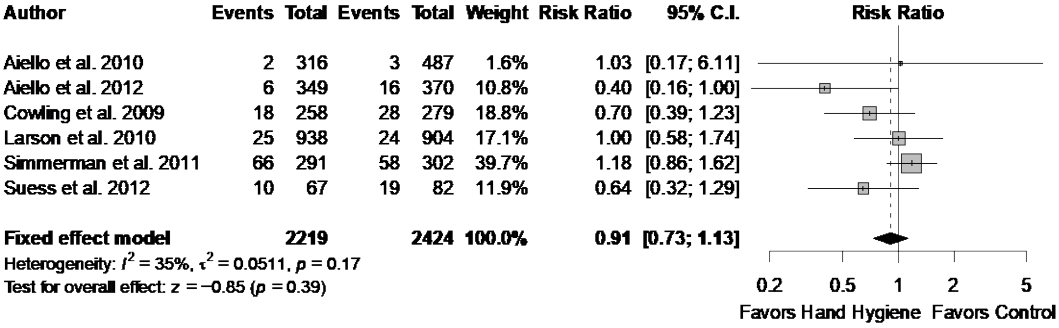

Hand hygiene with or without facemask

Author	Hand hygiene Events	Hand hygiene Total	Control Events	Control Total	Weight	Risk Ratio	95% C.I.
Aiello et al. 2010	2	316	3	487	0.5%	1.03	[0.17; 6.11]
Aiello et al. 2012	6	349	16	370	3.0%	0.40	[0.16; 1.00]
Cowling et al. 2008	5	84	12	205	1.3%	1.02	[0.37; 2.80]
Cowling et al. 2009	32	515	28	279	6.9%	0.62	[0.38; 1.01]
Larson et al. 2010	54	1884	24	904	6.2%	1.08	[0.67; 1.73]
Ram et al. 2015	17	177	10	250	1.6%	2.40	[1.13; 5.12]
Simmerman et al. 2011	132	583	58	302	14.6%	1.18	[0.89; 1.55]
Stebbins et al. 2011	51	1695	53	1665	10.2%	0.95	[0.65; 1.38]
Suess et al. 2012	10	67	19	82	3.3%	0.64	[0.32; 1.29]
Talaat et al. 2011	125	808	281	848	52.4%	0.47	[0.39; 0.56]

Heterogeneity: $I^2 = 82\%$, $\tau^2 = 0.2286$, $p < 0.01$

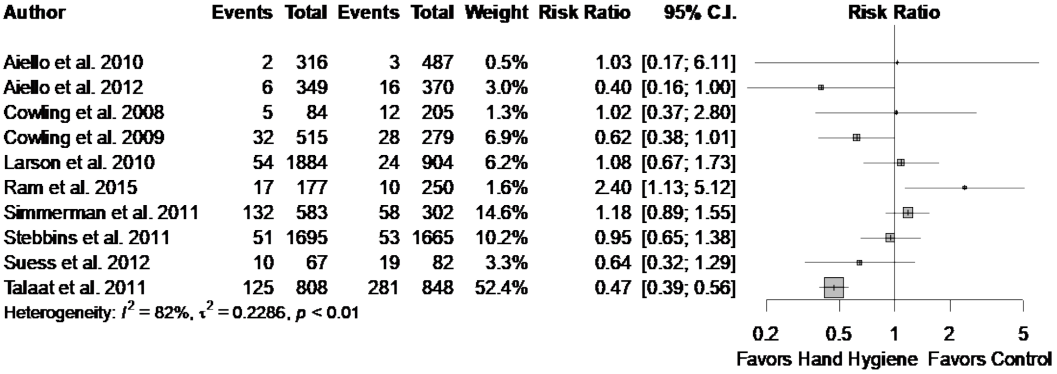

Appendix Figure 3. Metaanalysis of risk ratios for the effect of hand hygiene with or without face mask use on laboratory-confirmed influenza.

Hand hygiene only

Author	Hand hygiene Events	Hand hygiene Total	Control Events	Control Total	Weight	Risk Ratio	95% C.I.
Cowling et al. 2008	5	84	12	205	5.6%	1.02	[0.37; 2.80]
Cowling et al. 2009	14	257	28	279	21.7%	0.54	[0.29; 1.01]
Larson et al. 2010	29	946	24	904	19.8%	1.15	[0.68; 1.97]
Ram et al. 2015	17	177	10	250	6.7%	2.40	[1.13; 5.12]
Simmerman et al. 2011	66	292	58	302	46.1%	1.18	[0.86; 1.61]
**Fixed effect model**		**1756**		**1940**	**100.0%**	**1.11**	**[0.88; 1.39]**

Heterogeneity: $I^2 = 57\%$, $\tau^2 = 0.1119$, $p = 0.05$
Test for overall effect: $z = 0.89$ ($p = 0.38$)

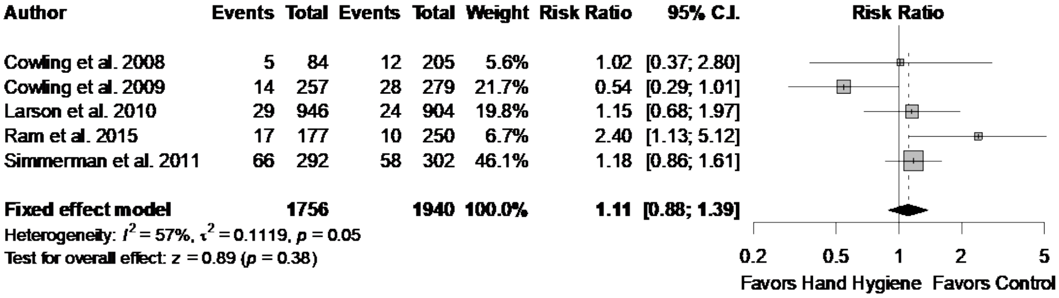

Hand hygiene and facemask

Author	Hand hygiene Events	Hand hygiene Total	Control Events	Control Total	Weight	Risk Ratio	95% C.I.
Cowling et al. 2009	18	258	28	279	21.5%	0.70	[0.39; 1.23]
Larson et al. 2010	25	938	24	904	19.5%	1.00	[0.58; 1.74]
Simmerman et al. 2011	66	291	58	302	45.4%	1.18	[0.86; 1.62]
Suess et al. 2012	10	67	19	82	13.6%	0.64	[0.32; 1.29]
**Fixed effect model**		**1554**		**1567**	**100.0%**	**0.97**	**[0.77; 1.22]**

Heterogeneity: $I^2 = 28\%$, $\tau^2 = 0.0263$, $p = 0.24$
Test for overall effect: $z = -0.27$ ($p = 0.79$)

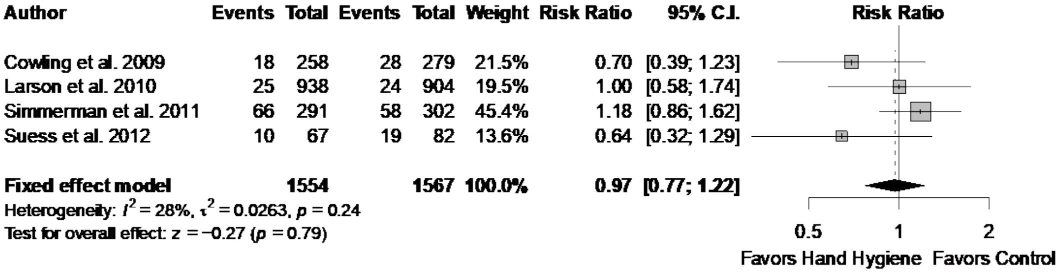

Hand hygiene with or without facemask

Author	Hand hygiene Events	Hand hygiene Total	Control Events	Control Total	Weight	Risk Ratio	95% C.I.
Cowling et al. 2008	5	84	12	205	3.9%	1.02	[0.37; 2.80]
Cowling et al. 2009	32	515	28	279	20.5%	0.62	[0.38; 1.01]
Larson et al. 2010	54	1884	24	904	18.3%	1.08	[0.67; 1.73]
Ram et al. 2015	17	177	10	250	4.7%	2.40	[1.13; 5.12]
Simmerman et al. 2011	132	583	58	302	43.0%	1.18	[0.89; 1.55]
Suess et al. 2012	10	67	19	82	9.6%	0.64	[0.32; 1.29]
**Fixed effect model**		**3310**		**2022**	**100.0%**	**1.05**	**[0.86; 1.27]**

Heterogeneity: $I^2 = 57\%$, $\tau^2 = 0.0950$, $p = 0.04$
Test for overall effect: $z = 0.46$ ($p = 0.65$)

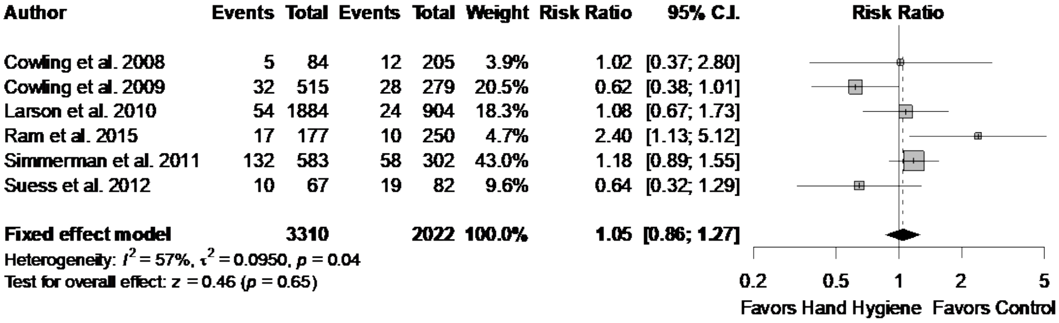

Appendix Figure 4. Metaanalysis of risk ratios for the effect of hand hygiene with or without face mask use on laboratory-confirmed influenza in household setting.

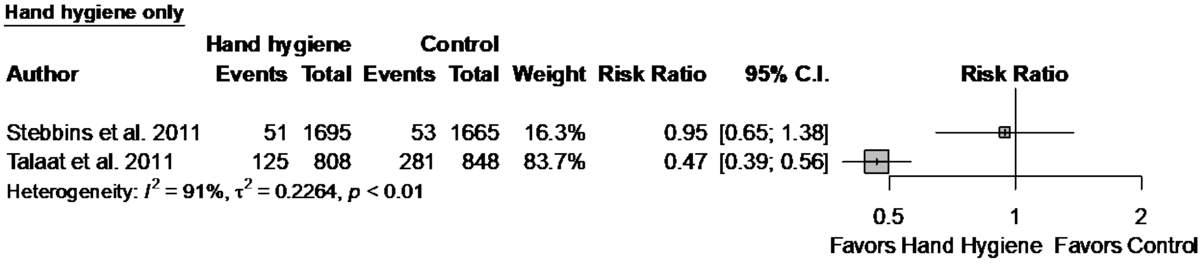

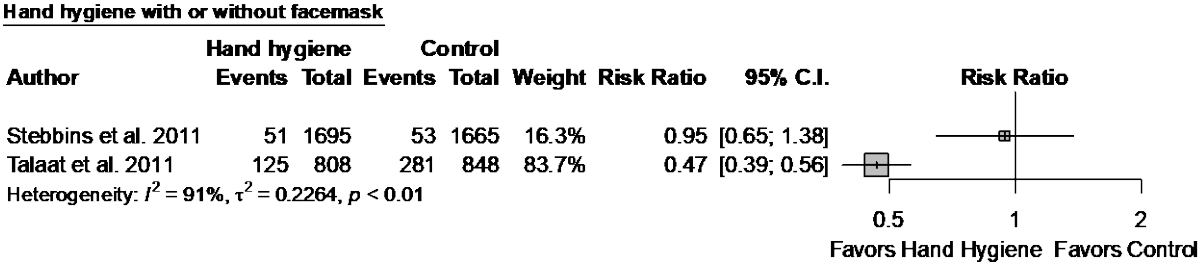

Appendix Figure 5. Metaanalysis of risk ratios for the effect of hand hygiene with or without face mask use on laboratory-confirmed influenza in elementary school setting.

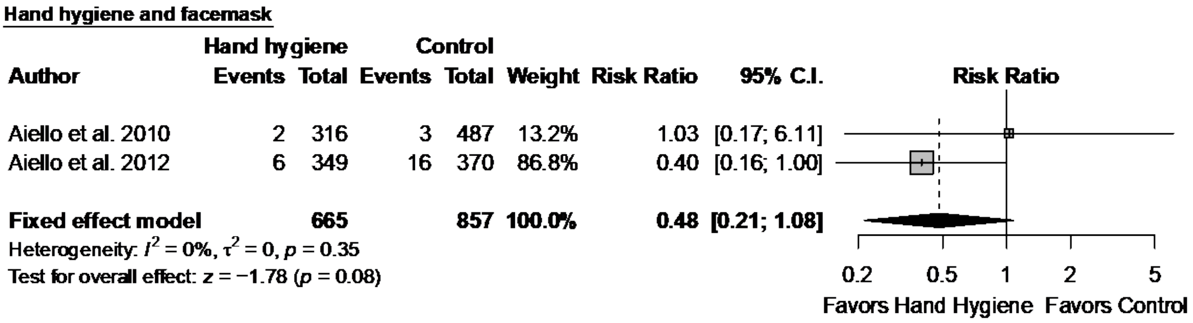

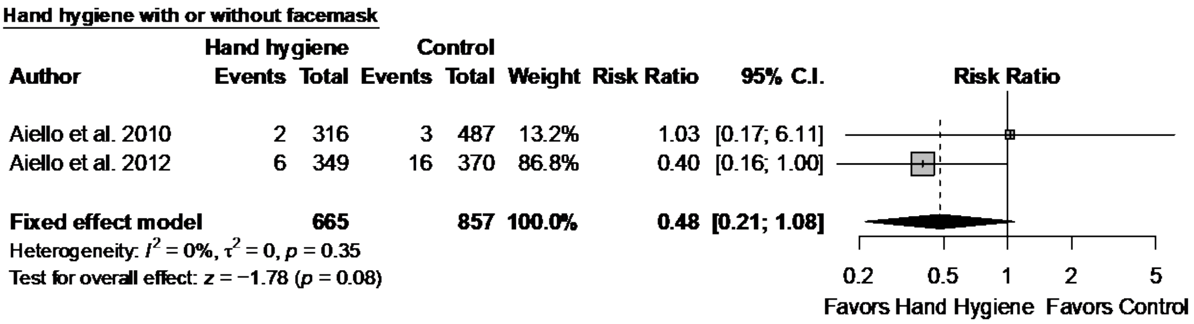

Appendix Figure 6. Metaanalysis of risk ratios for the effect of hand hygiene with or without face mask use on laboratory-confirmed influenza in university residence hall setting.

Appendix Table 4. Summary of studies included in the review of hand hygiene

Study	Study design	Study period	Population and setting	Transmission mode	Intervention	Outcome and finding
Azman AS, 2013 (8)	Cluster-RCT School level	Nov 2007–Apr 2008	3,360 students recruited from 10 elementary schools (Pittsburgh, USA)	Primary and secondary	Hand sanitizer, soap and education; control: receive no hand hygiene training	Primary transmission outcome refers to Stebbins, S. 2011 (9); no significant difference in secondary influenza-like-illness (ILI) attack rate between intervention group and control group
Levy JW, 2014 (10)	Cluster-RCT Household level	Jun 2009–Nov 2010	191 households with index children recruited from a public pediatric hospital (Bangkok, Thailand)	Secondary	Handwashing; handwashing and face mask ; control: receive no intervention	Fewer secondary influenza infections in households in the intervention group than control group, but not statistically significant; handwashing reduces surface influenza RNA contamination
Ram PK, 2015 (14)	Cluster-RCT Household level	Jan 2009–Dec 2010	384 households with index case-patients recruited from a hospital, among them, 60 index cases were laboratory-confirmed influenza infection (Kishoregoni, Bangladesh)	Secondary	Handwashing with soap and education; control: standard practice, no handwashing education	Handwashing promotion did not effectively prevent secondary influenza infection in household setting
Aiello AE, 2010 (18)	Cluster-RCT University residence hall level	Nov 2006–Mar 2007	1,437 university hall residents from 7 halls recruited, 1,297 residents were further analyzed (Michigan, USA)	Primary	Hand sanitizer, face mask and education; face mask and education; control: receive same education, but no additional intervention	The protective effect of interventions in reducing laboratory-confirmed influenza is not significant, but the interventions may be effective in ILI reduction
Aiello AE, 2012 (19)	Cluster-RCT University residence hall level	Nov 2007–Mar 2008	1,178 university hall residents recruited from 5 halls, 1,111 residents were further analyzed (Michigan, USA)	Primary	Hand sanitizer, face mask and education; face mask and education; control: receive same education, but no additional intervention	Reductions in the rates of influenza in the intervention groups, but results were statistically insignificant; combined intervention showed significant reduction in the rates of ILI
Cowling BJ, 2008 (12)	Cluster-RCT Household level	Feb 2007–Sep 2007	198 laboratory-confirmed influenza cases and their household contacts recruited from outpatient clinics (Hong Kong, China)	Secondary	Hand sanitizer and education; face mask and education; control: received same education but no additional interventions	No significant difference between intervention groups and control group in laboratory-confirmed influenza and clinical secondary attack rate
Cowling BJ, 2009 (11)	Cluster-RCT Household level	Jan 2008–Sep 2008	407 laboratory-confirmed influenza cases recruited from outpatient clinics, 259 households which included 794 household contacts were further analyzed (Hong Kong, China)	Secondary	Hand sanitizer and education; hand sanitizer, face mask and education; control: received same education but no additional interventions	Interventions prevent influenza transmission, but results were not statistically significant; combined intervention significantly reduce influenza transmission if implement within 36 h of symptom onset among index cases
Larson EL, 2010 (13)	Cluster-RCT Household level	Nov 2006–Jul 2008	617 households recruited, 509 households were further analyzed (New York, USA)	Primary	Hand sanitizer and education; hand sanitizer, face mask and education; control: receive same education,	No significant protective effect was detected of hand hygiene, or hand hygiene and face mask interventions on influenza prevention

Study	Study design	Study period	Population and setting	Transmission mode	Intervention	Outcome and finding
					but no additional intervention	
Simmerman JM, 2011 (15)	Cluster-RCT Household level	Apr 2008–Aug 2009	465 households recruited from a public pediatric hospital, 442 households were further analyzed (Bangkok, Thailand)	Secondary	Handwashing; handwashing and face mask ; control: received education that was unrelated to personal protective measures, but no additional interventions	Hand hygiene and face mask interventions did not reduce influenza transmission
Stebbins S, 2011 (9)	Cluster-RCT School level	Nov 2007–Apr 2008	3360 students recruited from 10 elementary schools (Pittsburgh, USA)	Primary	Hand sanitizer, soap and education; control: receive no hand hygiene training	"WHACK the Flu" programme did not reduce laboratory-confirmed influenza infection, but reduction of absence episodes and laboratory-confirmed influenza A infection was observed
Suess T, 2011 (16)	Cluster-RCT Household level	Nov 2009–Jan 2010 and Jan 2011–Apr 2011	84 laboratory-confirmed influenza cases and 218 household contacts recruited by general practitioners or pediatricians (Berlin, Germany)	Secondary	Hand sanitizer, face mask and infection prevention material; face mask and infection prevention material; control: receive same infection prevention material, but no additional intervention	The interventions could reduce influenza transmission in household setting if implemented early and used properly
Talaat M, 2011 (17)	Cluster-RCT School level	Feb 2008–May 2008	44,451 students recruited from 60 elementary schools (Cairo, Egypt)	Primary	Handwashing; control: receive no intervention	Hand hygiene campaign effectively reduced different kinds of infectious diseases, including laboratory-confirmed influenza

Appendix Table 5. GRADE quality assessment for hand hygiene

Quality assessment							No. patients		Effect		
No. of studies	Design	Risk for bias	Inconsistency	Indirectness	Imprecision	Other considerations	Hand hygiene with or without face mask versus control		Risk ratio	Quality	Importance
Effect of hand hygiene intervention on prevention of laboratory-confirmed influenza											
10	Randomized trial[1,2]	No serious risk for bias[3–7]	Serious inconsistency[8]	Serious indirectness[9]	No serious imprecision[10]	None	434/6478 (6.7%)	504/5392 (9.3%)	Risk ratio cannot be generated because of high heterogeneity	Low	Important

[1]All studies were randomized trials.
[2]All studies were cluster-RCTs: six studies at household level, two studies at school level and two studies at university residence level.
[3]Five studies reported blinding of study staff including clinical staff, laboratory staff or recruiting physicians. Subjects of all studies were not blinded due to the nature of the study design.
[4]Three studies used block randomization and seven studies used simple randomization.
[5]Allocation concealment was adequate in all trials. Nine studies described the baseline characteristics of participants in all intervention groups. No serious baseline imbalance was observed.
[6]All studies reported the number of loss to follow-up in all intervention groups. No serious differential loss to follow-up occurred for whole clusters or individuals in a cluster.
[7]All studies adjusted for clustering in their analysis.
[8]High heterogeneity was observed in the pooled analysis (I^2 > 50%).
[9]Studies evaluating the combined intervention were included.
[10]Total sample size is sufficient for a single adequately powered study.

Respiratory Etiquette

Terminology

Relevant terminology relating to respiratory etiquette is shown as follows (Appendix Table 6):

Appendix Table 6. Definition of terms relevant to respiratory etiquette

Term	Definition
Respiratory etiquette	Respiratory etiquette is also known as 'cough etiquette' (*20*). It is a simple hygiene practice to prevent person-to-person transmission of respiratory infections. Measures include (*21*):<br>1. Cover the mouth and nose with a tissue or mask when coughing or sneezing<br>2. Dispose the used tissue or mask in the nearest waste basket immediately<br>3. Proper hand hygiene after touching respiratory secretions and/or contaminated objects

Search Strategy

We conducted a literature search on 6 November 2018 using the following search terms (Appendix Table 7) in 4 databases (PubMed, Medline, EMBASE, and CENTRAL) to identify literatures that were available from 1946 through November 5, 2018. Studies were selected if they investigated specifically the use of respiratory/cough etiquette as the intervention along with the study outcome of laboratory-confirmed influenza virus infection. Studies that reported use of face mask as part of the respiratory etiquette were excluded because they will be covered in the next section. We reviewed literatures of all languages. Two reviewers (E.S. and S.G.) reviewed retrieved titles and subsequent relevant abstracts independently. Titles and abstract selected by any of the reviewers were included for subsequent screening. Both reviewers reviewed full-text and extracted data for selected studies independently. If a consensus was not reached, further discussion was held or opinion was obtained from a third reviewer.

Appendix Table 7. Search strategy for respiratory etiquette

Search terms	Search date	Reviewers
#1: "respiratory hygiene" OR "cough etiquette" OR "respiratory etiquette"<br>#2: "influenza" OR "flu"<br>#3: #1 AND #2	November 6, 2018	E.S., H.G.

Findings

Eighty articles were retrieved from 4 electronic databases after removing duplicate publications. A total of 35 abstracts were selected for screening and 18 full-text articles were assessed for eligibility. No studies were identified for this review to quantify the efficacy of respiratory etiquette with the outcome of laboratory-confirmed influenza. The flowchart is shown in Appendix Figure 7.

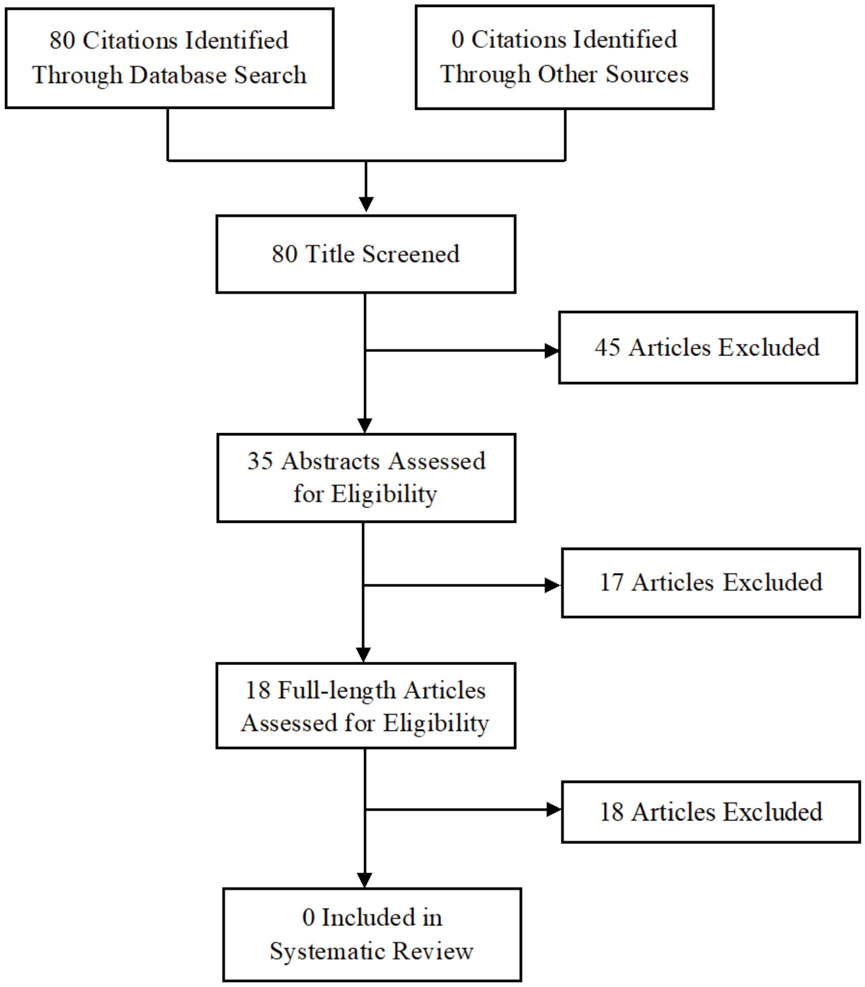

Appendix Figure 7. Flowchart of literature search and study selection for respiratory etiquette.

Face Masks

Terminology

Relevant terminology relating to face masks are shown as follows (Appendix Table 8):

Appendix Table 8. Definition of terms relevant to face masks

Types of masks	Terminology
Clothing, scarf, or rags tied over the nose and mouth	These are referred as alternative barriers to face mask s, but there is insufficient information available on their effectiveness on disease prevention (*22*).
Cloth mask	Cloth masks can be referred to “reusable masks made of cloth or any other fabric, including cotton, silk or muslin” (*23*). Filtration capacity is determined by the fitness of fabric and number of layers of a cloth mask (*23*). Cloth masks should be cleaned with household detergent thoroughly between each use (*24*).
Face mask	A face mask , also known as surgical, isolation, dental or medical procedure masks, is a loose-fitting, single-use disposable device that covers the mouth and nose of the user, and helps block large-particle droplets, splashes, sprays or splatter that may contain infectious agents (*25*). Face mask s may also help reduce exposure of user’s saliva and respiratory secretions to others (*25*). They are not designed to protect against breathing in small-particle aerosols that may contain viruses.
Respirator	Respirator, also known as filtering facepiece respirator (FFR), is a personal protective device that covers the nose and mouth of the user, and helps reduce the risk for inhaling hazardous airborne particles (including dust particles and infectious agents), gases, or vapors on the user (*26*).

Types of masks	Terminology
	The National Institute for Occupational Safety and Health (NIOSH) in the United States certifies N, R and P series particulate filtering respirator types 95, 99 and 100 with minimum filtration efficiencies of 95, 99 and 99.97%, respectively. This certification is recognized by countries such as Canada, Mexico, and Chile. In Europe, respirators marked with 'Conformité Européen' (CE) such as FFP1 (class P1), FFP2 (class P2) and FFP3 (class P3) types meet minimum filtration efficiencies of 80, 94 and 99%, respectively (*27*).

Search Strategy

We conducted a literature search on July 28, 2018 by using the following search terms (Appendix Table 9) in 4 databases (PubMed, Medline, EMBASE, and CENTRAL) to identify literatures that were available from 1946 through July 26, 2018. Studies were selected if they were conducted in randomized controlled trial in community settings, such as households and schools, evaluated the use of face masks with or without the combination of other intervention as 1 intervention and included the incidence of laboratory-confirmed influenza case as a study outcome. We reviewed literatures of all languages. Two reviewers (E.S. and J.X.) reviewed retrieved titles and subsequent relevant abstracts independently. Titles and abstract selected by any of the reviewers were included for subsequent screening. Both reviewers reviewed full-text and extracted data for selected studies independently. If a consensus was not reached, further discussion was held or opinion was obtained from a third reviewer.

Appendix Table 9. Search strategy for face masks

Search terms	Search date	Reviewers
#1: "face mask " OR "face masks" OR "mask" OR "masks" OR "respirator" OR "respirators"<br>#2: "influenza" OR "flu"<br>#3: #1 AND 2	July 28, 2018	E.S., J.X.

Findings

A total of 1,100 articles were retrieved from four electronic databases after removing duplicate records. Ten relevant studies were identified for this review and metaanalysis to quantify the efficacy of community-based use of face mask s after excluding 89 articles by full-text assessment (Appendix Table 10). The flowchart is shown in Appendix Figure 8.

A total of 7/10 studies were conducted in household settings (*11–13*,*15*,*16*,*28*,*29*), with 2 studies conducted in university residential halls (*18*,*19*), and 1 study was conducted in Hajj pilgrims (*28*). Nearly half of the studies evaluated the effect of face mask use with the practice of hand hygiene, therefore results were analyzed in 2 groups *1*) comparison of control group with intervention group of face mask use only, and *2*) comparison of control group with intervention group of face mask use with or without hand hygiene (Appendix Figure 9).

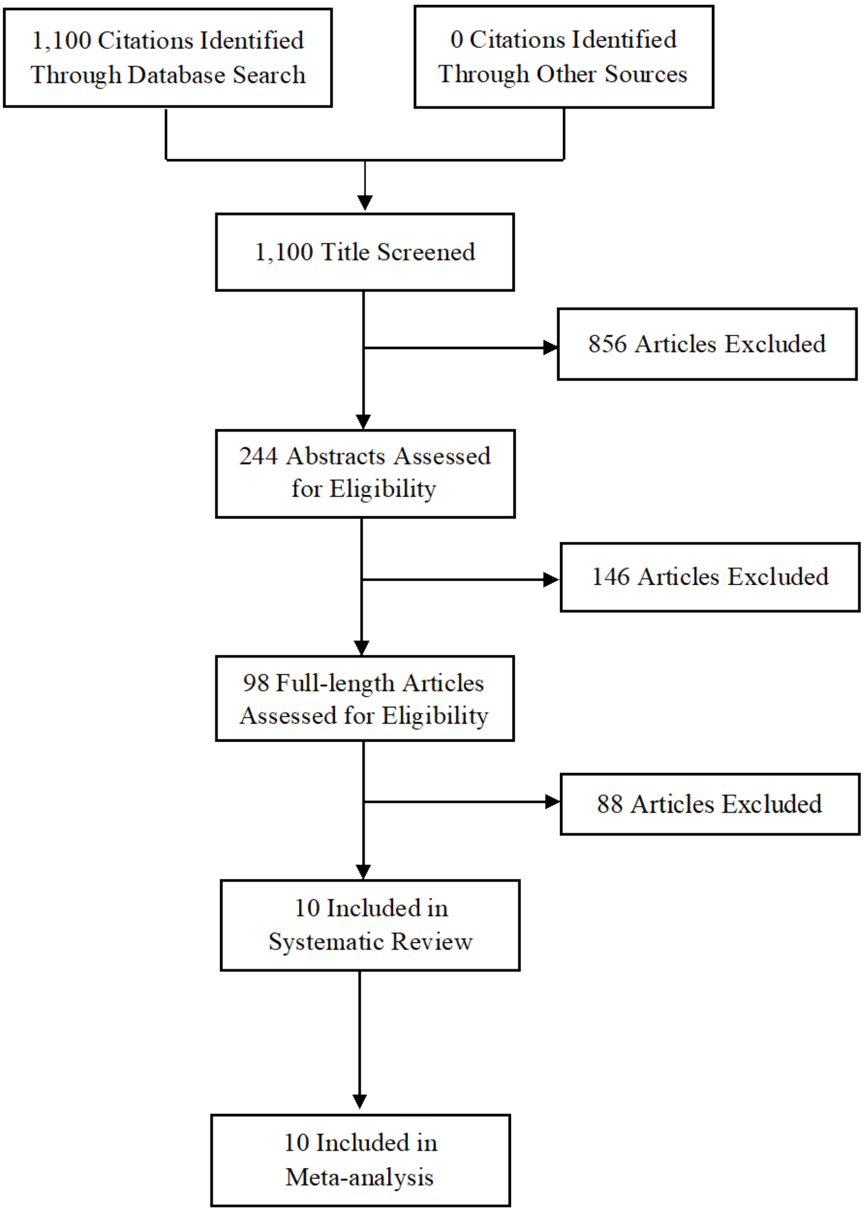

Appendix Figure 8. Flowchart of literature search and study selection for face masks.

Among the 10 selected studies, two studies by MacIntyre et al. had a slightly different study design. One study enrolled families in which one person had laboratory-confirmed influenza, and only required the household contacts to wear face masks or P2 masks (equivalent to a N95 respirator) (*28*), whereas another study required only the ill members to wear face masks to evaluate the protective effect of face mask if worn by the ill individual (i.e., source control) (*29*). In the remaining 8 studies, every participant in the face mask intervention group was supposed to wear a face mask.

MacIntyre et al. compared the protective effect of face mask and P2 mask but they found no significant difference in ILI and laboratory-confirmed respiratory infections (influenza A and B virus, RSV, hMPV, adenovirus, PIV, coronavirus, rhinovirus, enterovirus, picornovirus); however, they reported a significant reduction in ILI if the mask was worn with good compliance in a secondary analysis (*28*).

Two studies by Aiello et al. were conducted in residential hall settings evaluating the effectiveness of face masks as a primary protection (*18*,*19*). They randomized university residents by cluster (each residential hall forming a cluster unit) to face masks, enhanced hand hygiene, or both. They then measured the incidence of laboratory-confirmed influenza in students in each hall. They reported no significant difference in ILI and laboratory-confirmed influenza in these three randomized groups; however, they observed a significant reduction in ILI in the combined face mask and hand hygiene intervention group during the latter half of the study period in a secondary analysis.

Seven studies were conducted in household settings where a person with laboratory-confirmed influenza was recruited as a household index case and the rate of secondary infections in the education group (control), mask group or hand hygiene group was monitored for illnesses and infections (*11–13*,*15*,*16*,*28*,*29*). All studies found no significant differences in the rate of laboratory-confirmed influenza virus infections in contacts in the face mask arms, and some studies reported that low compliance of the use of NPIs could affect the results (*13*). One study reported a significant reduction in laboratory-confirmed influenza virus infections in contacts in the face mask and hand hygiene group in the subset of households where the intervention was applied within 36 hours of symptom onset in the index case (*16*).

Ten studies were pooled to conduct a metaanalysis to quantify the efficacy of community-based use of face masks in the reduction of laboratory-confirmed influenza virus infection (*11–13*,*15–19*,*28–30*). In the pooled analysis, there was a nonsignificant RR reduction of 22% (RR 0.78, 95% CI 0.51–1.20; I^2 = 30%, p = 0.25) in the face mask group and 8% in the face mask group regardless of the enhanced hand hygiene (RR 0.92, 95% CI 0.75–1.12; I^2 = 30%, p = 0.40) (Appendix Figure 9).

Appendix Table 11 shows the results of quality assessment of evidence on face mask intervention by using the GRADE approach.

Mask only

Author	Mask Events	Mask Total	Control Events	Control Total	Weight	Risk Ratio	95% C.I.
Aiello et al. 2010	5	347	3	487	5.7%	2.34	[0.56; 9.72]
Aiello et al. 2012	12	392	16	370	37.3%	0.71	[0.34; 1.48]
Barasheed et al. 2014	1	11	0	28	0.7%	7.43	[0.33; 169.47]
Cowling et al. 2008	4	61	12	205	12.5%	1.12	[0.37; 3.35]
MacIntyre et al. 2009	1	94	0	100	1.1%	3.19	[0.13; 77.36]
MacIntyre et al. 2016	0	302	1	295	3.4%	0.33	[0.01; 7.96]
Suess et al. 2012	6	69	19	82	39.4%	0.38	[0.16; 0.89]
**Fixed effect model**		**1276**		**1567**	**100.0%**	**0.78**	**[0.51; 1.20]**

Heterogeneity: $I^2 = 30\%$, $\tau^2 = 0.1899$, $p = 0.20$
Test for overall effect: $z = -1.15$ ($p = 0.25$)

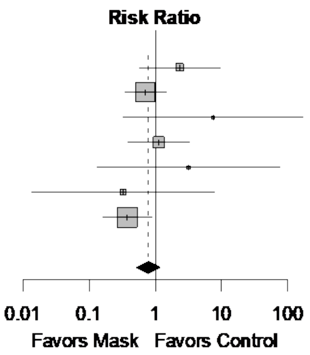

Facemask and hand hygiene

Author	Mask Events	Mask Total	Control Events	Control Total	Weight	Risk Ratio	95% C.I.
Aiello et al. 2010	2	316	3	487	1.6%	1.03	[0.17; 6.11]
Aiello et al. 2012	6	349	16	370	10.8%	0.40	[0.16; 1.00]
Cowling et al. 2009	18	258	28	279	18.8%	0.70	[0.39; 1.23]
Larson et al. 2010	25	938	24	904	17.1%	1.00	[0.58; 1.74]
Simmerman et al. 2011	66	291	58	302	39.7%	1.18	[0.86; 1.62]
Suess et al. 2012	10	67	19	82	11.9%	0.64	[0.32; 1.29]
**Fixed effect model**		**2219**		**2424**	**100.0%**	**0.91**	**[0.73; 1.13]**

Heterogeneity: $I^2 = 35\%$, $\tau^2 = 0.0511$, $p = 0.17$
Test for overall effect: $z = -0.85$ ($p = 0.39$)

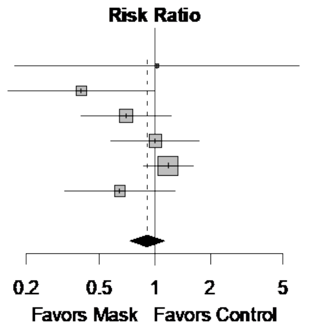

Facemask with or without hand hygiene

Author	Mask Events	Mask Total	Control Events	Control Total	Weight	Risk Ratio	95% C.I.
Aiello et al. 2010	7	663	3	487	2.1%	1.71	[0.45; 6.59]
Aiello et al. 2012	18	741	16	370	13.0%	0.56	[0.29; 1.09]
Barasheed et al. 2014	1	11	0	28	0.2%	7.43	[0.33; 169.47]
Cowling et al. 2009	18	258	28	279	16.3%	0.70	[0.39; 1.23]
Cowling et al. 2008	4	61	12	205	3.3%	1.12	[0.37; 3.35]
Larson et al. 2010	25	938	24	904	14.9%	1.00	[0.58; 1.74]
MacIntyre et al. 2009	1	94	0	100	0.3%	3.19	[0.13; 77.36]
MacIntyre et al. 2016	0	302	1	295	0.9%	0.33	[0.01; 7.96]
Simmerman et al. 2011	66	291	58	302	34.6%	1.18	[0.86; 1.62]
Suess et al. 2012	16	136	19	82	14.4%	0.51	[0.28; 0.93]
**Fixed effect model**		**3495**		**3052**	**100.0%**	**0.92**	**[0.75; 1.12]**

Heterogeneity: $I^2 = 30\%$, $\tau^2 = 0.0593$, $p = 0.17$
Test for overall effect: $z = -0.84$ ($p = 0.40$)

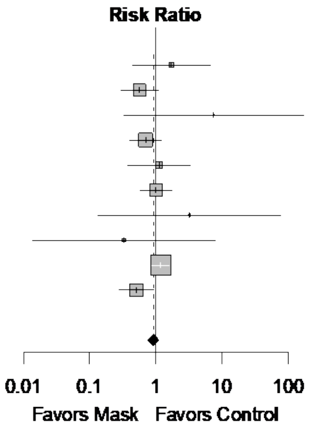

Appendix Figure 9. Metaanalysis of risk ratios for the effect of face mask use with or without enhanced hand hygiene on laboratory-confirmed influenza.

Appendix Table 10. Summary of studies included in the review of face masks

Study	Study design	Study period	Population and setting	Intervention	Outcome and finding
Aiello AE, 2010 (18)	Cluster-RCT University residence hall level	Nov 2006–Mar 2007	1437 university hall residents recruited, 1297 residents were further analyzed (Michigan, USA)	Hand sanitizer and face mask and education; face mask and education; control received the same education because all intervention groups but no additional interventions were given	Significant reduction in ILI in the latter half of the study period in mask and hand hygiene group compared with the control but no significant reduction in ILI in mask and hand group or mask-only group or control
Aiello AE, 2012(19)	Cluster-RCT University residence hall level	Nov 2007–Mar 2008	1,178 university hall residents recruited from 5 halls, 1,111 residents were further analyzed (Michigan, USA)	Hand sanitizer and face mask and education; face mask and education; control received the same education because all intervention groups but no additional interventions were given	No significant reduction in rates of laboratory-confirmed influenza in mask and hand group or mask-only group or control group
Barasheed O, 2014 (30)	Cluster-RCT Hajj pilgrimage	Nov 2011–Nov 2011	164 Australian pilgrims recruited from 2011 Hajj (Saudi Arabia)	Face mask; control were not provided with face mask s during the study period	No significant difference in laboratory-confirmed influenza between control and mask-only group but protective effect was observed against syndromic ILI in mask-only group compared with the control (31% vs. 53%, p = 0.04)
Cowling BJ, 2008 (12)	Cluster-RCT Household level	Feb 2007–Sep 2007	198 laboratory-confirmed influenza cases and their household contacts recruited from outpatient clinics (Hong Kong, China)	Hand sanitizer and education; face mask and education; control received same education because all intervention groups but no additional interventions were given	No significant reduction in the secondary influenza attack rate in control, mask or hand group
Cowling BJ, 2009 (11)	Cluster-RCT Household level	Jan 2008–Sep 2008	407 laboratory-confirmed influenza cases recruited from outpatient clinics, 259 households which included 794 household contacts were further analyzed (Hong Kong, China)	Hand sanitizer and education; hand sanitizer, face mask and education; control received same education because all intervention groups but no additional interventions were given	No significant difference in rates of laboratory-confirmed influenza in control, hand-only or mask and hand group
Larson EL, 2010 (13)	Cluster-RCT Household level	Nov 2006–Jul 2008	617 households recruited, 509 households were further analyzed (New York, NY, USA)	Hand sanitizer and education; hand sanitizer, face mask and education; control received same education because all intervention groups but no additional interventions were given	No significant reduction in rates of laboratory-confirmed nfluenza in control, hand-only, mask or hand group
MacIntyre CR, 2009 (28)	Cluster-RCT Household level	Aug 2006–Oct 2006 and Jun 2007–Oct 2007	145 laboratory-confirmed influenza cases and their adult household contacts recruited from a pediatric health service (Sydney, Australia)	Surgical mask; P2 mask; control were not provided with any masks during the study period	No significant difference in rate of laboratory confirmed influenza in control, face mask-only or P2 mask-only group
MacIntyre CR, 2016 (29)	Cluster-RCT Household level	Nov 2013–Jan 2014	245 ILI cases and 597 household contacts recruited from fever clinics	Face mask; control were not provided with any masks during the study period	Clinical respiratory illness, ILI and laboratory-confirmed viral infections were lower in the mask-only group

Study	Study design	Study period	Population and setting	Intervention	Outcome and finding
			(Beijing, China)		compared with the control group, but results were not statistically significant
Simmerman JM, 2011) (15)	Cluster-RCT Household level	Apr 2008–Aug 2009	465 households recruited from a public pediatric hospital, 442 households were further analyzed (Bangkok, Thailand)	Handwashing; handwashing and face mask; control received education that was unrelated to personal protective measures and no additional interventions were given	No significant reduction in rate of secondary influenza infection in control, hand-only, mask or hand group
Suess (2012) (16)	Cluster-RCT Household level	Nov 2009–Jan 2010 and Jan 2011–Apr 2011	84 laboratory-confirmed influenza cases and 218 household contacts recruited by general practitioners or pediatricians (Berlin, Germany)	Hand sanitizer and face mask; face mask; control were not provided with any face masks nor hand-rub during the study period	No significant difference in rate of laboratory confirmed influenza in control, mask-only, mask or hand group

Appendix Table 11. GRADE quality assessment for face masks

Quality assessment							No. of patients		Effect		
No. studies	Design	Risk for bias	Inconsistency	Indirectness	Imprecision	Other considerations	Face mask with or without hand hygiene versus control		Risk ratio (95% CI)	Quality	Importance
Effect of face mask intervention on prevention of laboratory-confirmed influenza											
10	Randomized trial[1,2]	No serious risk for bias[3–7]	Serious inconsistency[8]	Serious indirectness[9]	No serious imprecision[10]	None	156/3495	161/3052	0.92 (0.75–1.12)	Low	Important

[1]All studies were randomized trials.
[2]All studies were cluster-RCTs: two studies at university residence level, seven studies at household level and one study randomized by sleeping tent during Hajj pilgrim.
[3]Eight studies reported blinding of study staffs including clinical staff, laboratory staff or recruiting physicians. Subjects of all studies were not blinded.
[4]Three studies used block randomization; six studies used computer program to generate the randomization order and one study used ticket-picking for selection.
[5]Allocation concealment was adequate in all trials. Eight studies described the baseline characteristics of participants in all intervention groups. No serious baseline imbalance was observed.
[6]All study reported the number of loss to follow-up in all intervention groups. No serious differential loss to follow-up occurred for whole clusters or persons in a cluster.
[7]Seven studies adjusted for clustering in their analysis.
[8]Moderate heterogeneity was observed in the pooled analysis.
[9]Studies evaluating the combined intervention were included.
[10]Total sample size is insufficient in the pooled analysis.

Surface and Object Cleaning

Terminology

Relevant terminology relating to surface and object cleaning is shown as follows (Appendix Table 12):

Appendix Table 12. Definition of terms relevant to surface and object cleaning

Term	Definition
Surface and object cleaning	Routine cleaning of frequently used surfaces and objects to reduce influenza transmission.

Search Strategy

We conducted a literature search on October 15, 2018 by using the following search terms (Appendix Table 13) in 4 databases (PubMed, Medline, EMBASE, and CENTRAL) to identify literature that was available from 1946 through October 14, 2018. Study selection criteria are studies reporting the effect of surface and object cleaning intervention compared with no intervention in preventing influenza virus infections in community settings. There were no limitations on the types of cleaning techniques. Randomized controlled trials and other types of epidemiologic studies were included if they evaluated the effect of surface and object cleaning on laboratory-confirmed influenza, ILI or respiratory illness. Simulation studies, recommendations, and commentaries/editorials were excluded. We reviewed literatures of all languages. Two reviewers (J.X. and E.S.) reviewed retrieved titles and subsequent relevant abstracts independently. Titles and abstract selected by any of the reviewers were included for subsequent screening. Both reviewers reviewed full-text and extracted data for selected studies independently. If a consensus was not reached, further discussion was held or opinion was obtained from a third reviewer.

Appendix Table 13. Search strategy for surface and object cleaning

Search terms	Search date	Reviewers
#1: "surface" OR "surfaces" OR "object" OR "objects" OR "fomite" OR "fomites" OR "environment" OR "environmental"<br>#2: "clean" OR "cleans" OR "cleaning" OR "cleanse" OR "cleansing" OR "disinfect" OR "disinfects" OR "disinfection" OR "disinfecting" OR "wipe" OR "wipes" OR "sanitize" OR "sanitizes" OR "sanitizing" OR "sanitation" OR "sterilize" OR "sterilizes" OR "sterilizing" OR "sterilization" OR "sterilise" OR "sterilises" OR "sterilising" OR "sterilisation" OR "decontaminate" OR "decontaminates" OR "decontaminating" OR "decontamination"<br>#3: "influenza" OR "flu"<br>#4: #1 AND #2 AND #3	October 15, 2018	J.X., E.S.

Findings

We identified 484 reviews through the search, of which 462 reviews were removed during title and abstract screening. We further excluded 19 articles after full text assessment because

they did not specify the surface or object cleaning as the study intervention or respiratory infections as the study outcome. Three articles were included in the systematic review to study the effectiveness of surface and object cleaning to prevent influenza infection. The flowchart is shown in Appendix Figure 10.

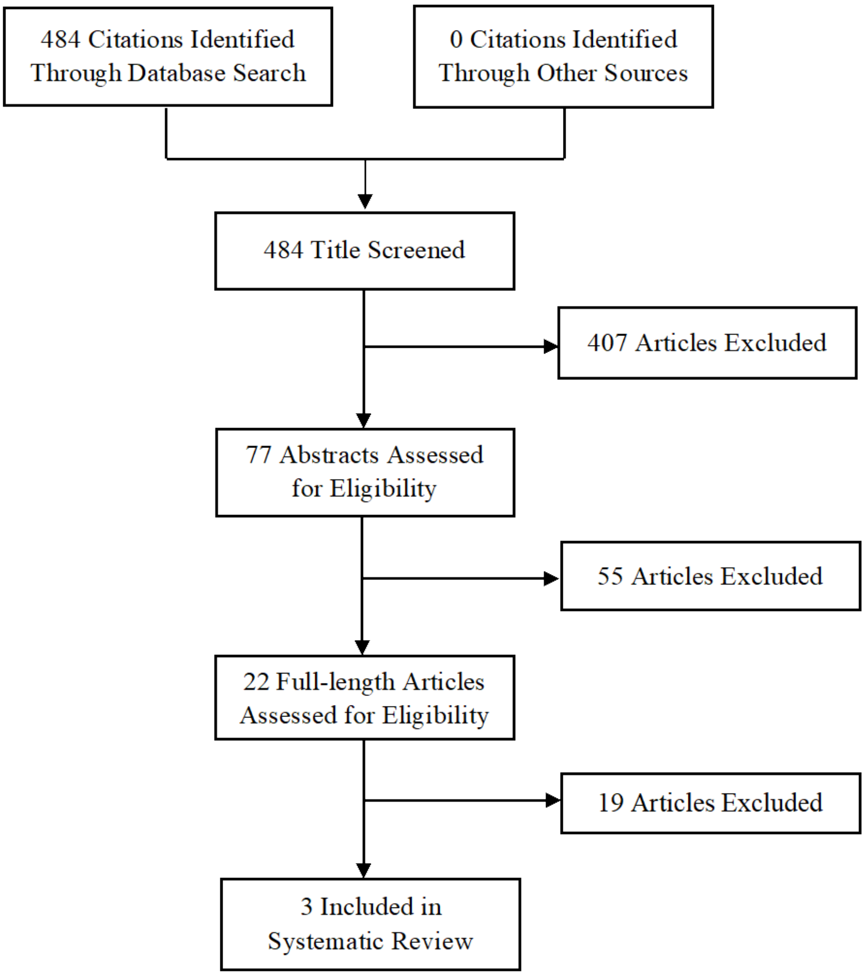

Appendix Figure 10. Flowchart of literature search and study selection for surface and object cleaning.

A cross-sectional study showed that bleach use in households was associated with a statistically significant increase in self-reported influenza based on self-administered questionnaires. The authors, however, did not specify the definition of influenza illness and they also hypothesized that the increase of cases might be due to the immunosuppressive properties of bleach (*31*). A randomized controlled trial with disinfection of toys and linen in day care nurseries reported a reduction in the detections of viruses in the environment, but no significant

reduction was observed on influenza-related and other acute respiratory-related illnesses among children (*32*). Another randomized controlled trial conducted in elementary schools demonstrated that hand hygiene with alcohol-based hand sanitizer and surface disinfection with quaternary ammonium wipes intervention could reduce gastrointestinal illness absenteeism, but not respiratory illness absenteeism (*32*). Detailed study description is shown in Appendix Table 14.

Appendix Table 14. Summary of studies included in the review of surface and object cleaning

Study	Study design	Study period	Population and setting	Intervention	Outcome measures	Main findings
Casas L, 2015 (30)	Cross-sectional study	Apr 2008–Dec 2010	9,102 students from schools (Spain, Netherlands and Finland)	Not intervention. Environment cleaning with bleach versus no use of bleach	Self-reported influenza	Passive contact with cleaning bleach in the household might increase the risk for respiratory and other infections in children, which might have an adverse effect on school-age children's health
Ibfelt T, 2015 (31)	Cluster-RCT	Autumn 2012–Apr 2013	12 d-care nurseries caring for 587 children (Copenhagen, Denmark)	Disinfection of toys; control: receive no intervention	Respiratory infections and surface sample influenza virus detection	Frequent disinfection of toys could reduce the presence of environmental microbial, but not significantly reduce respiratory illness of nursery children
Sandora TJ, 2008 (32)	Cluster-RCT	Mar 2006–May 2006	285 students from elementary schools (Ohio, USA)	Hand hygiene and surface cleaning; control: usual baseline practice, no additional intervention	Respiratory illness	Surface disinfection could reduce gastrointestinal-related absenteeism among school-age children, but not respiratory-related illness

References

1. World Health Organization. WHO guidelines on hand hygiene in health care. Report No.: WHO/IER/PSP/2009.07. Geneva: The Organization; 2009.

2. Boyce JM, Pittet D; Healthcare Infection Control Practices Advisory Committee; HICPAC/SHEA/APIC/IDSA Hand Hygiene Task Force; Society for Healthcare Epidemiology of America/Association for Professionals in Infection Control/Infectious Diseases Society of America. Guideline for Hand Hygiene in Health-Care Settings. Recommendations of the Healthcare Infection Control Practices Advisory Committee and the HICPAC/SHEA/APIC/IDSA Hand Hygiene Task Force. MMWR Recomm Rep. 2002;51(RR-16):1–45, quiz CE1–4. PubMed

3. Chughtai AA, Seale H, MacIntyre CR. Use of cloth masks in the practice of infection control—evidence and policy gaps. Int J Infect Control. 2013;9:1–12. https://doi.org/10.3396/IJIC.v9i3.020.13

4. McGuinness SL, Barker SF, O'Toole J, Cheng AC, Forbes AB, Sinclair M, et al. Effect of hygiene interventions on acute respiratory infections in childcare, school and domestic settings in low- and middle-income countries: a systematic review. Trop Med Int Health. 2018;23:816–33. PubMed https://doi.org/10.1111/tmi.13080

5. Saunders-Hastings P, Crispo JAG, Sikora L, Krewski D. Effectiveness of personal protective measures in reducing pandemic influenza transmission: a systematic review and meta-analysis. Epidemics. 2017;20:1–20. PubMed https://doi.org/10.1016/j.epidem.2017.04.003

6. Warren-Gash C, Fragaszy E, Hayward AC. Hand hygiene to reduce community transmission of influenza and acute respiratory tract infection: a systematic review. Influenza Other Respir Viruses. 2013;7:738–49. PubMed https://doi.org/10.1111/irv.12015

7. Wong VW, Cowling BJ, Aiello AE. Hand hygiene and risk of influenza virus infections in the community: a systematic review and meta-analysis. Epidemiol Infect. 2014;142:922–32. PubMed https://doi.org/10.1017/S095026881400003X

8. Azman AS, Stark JH, Althouse BM, Vukotich CJ Jr, Stebbins S, Burke DS, et al. Household transmission of influenza A and B in a school-based study of non-pharmaceutical interventions. Epidemics. 2013;5:181–6. PubMed https://doi.org/10.1016/j.epidem.2013.09.001

9. Stebbins S, Cummings DA, Stark JH, Vukotich C, Mitruka K, Thompson W, et al. Reduction in the incidence of influenza A but not influenza B associated with use of hand sanitizer and cough hygiene in schools: a randomized controlled trial. Pediatr Infect Dis J. 2011;30:921–6. PubMed https://doi.org/10.1097/INF.0b013e3182218656

10. Levy JW, Suntarattiwong P, Simmerman JM, Jarman RG, Johnson K, Olsen SJ, et al. Increased hand washing reduces influenza virus surface contamination in Bangkok households, 2009–2010. Influenza Other Respir Viruses. 2014;8:13–6. PubMed https://doi.org/10.1111/irv.12204

11. Cowling BJ, Chan K-H, Fang VJ, Cheng CK, Fung RO, Wai W, et al. Facemasks and hand hygiene to prevent influenza transmission in households: a cluster randomized trial. Ann Intern Med. 2009;151:437–46. PubMed https://doi.org/10.7326/0003-4819-151-7-200910060-00142

12. Cowling BJ, Fung RO, Cheng CK, Fang VJ, Chan KH, Seto WH, et al. Preliminary findings of a randomized trial of non-pharmaceutical interventions to prevent influenza transmission in households. PLoS One. 2008;3:e2101. PubMed https://doi.org/10.1371/journal.pone.0002101

13. Larson EL, Ferng YH, Wong-McLoughlin J, Wang S, Haber M, Morse SS. Impact of non-pharmaceutical interventions on URIs and influenza in crowded, urban households. Public Health Rep. 2010;125:178–91. PubMed https://doi.org/10.1177/003335491012500206

14. Ram PK, DiVita MA, Khatun-e-Jannat K, Islam M, Krytus K, Cercone E, et al. Impact of intensive handwashing promotion on secondary household influenza-like illness in rural Bangladesh: findings from a randomized controlled trial. PLoS One. 2015;10:e0125200. PubMed https://doi.org/10.1371/journal.pone.0125200

15. Simmerman JM, Suntarattiwong P, Levy J, Jarman RG, Kaewchana S, Gibbons RV, et al. Findings from a household randomized controlled trial of hand washing and face masks to reduce influenza transmission in Bangkok, Thailand. Influenza Other Respir Viruses. 2011;5:256–67. PubMed https://doi.org/10.1111/j.1750-2659.2011.00205.x

16. Suess T, Remschmidt C, Schink SB, Schweiger B, Nitsche A, Schroeder K, et al. The role of face mask s and hand hygiene in the prevention of influenza transmission in households: results from a cluster randomised trial; Berlin, Germany, 2009–2011. BMC Infect Dis. 2012;12:26. PubMed https://doi.org/10.1186/1471-2334-12-26

17. Talaat M, Afifi S, Dueger E, El-Ashry N, Marfin A, Kandeel A, et al. Effects of hand hygiene campaigns on incidence of laboratory-confirmed influenza and absenteeism in schoolchildren, Cairo, Egypt. Emerg Infect Dis. 2011;17:619–25. PubMed https://doi.org/10.3201/eid1704.101353

18. Aiello AE, Murray GF, Perez V, Coulborn RM, Davis BM, Uddin M, et al. Mask use, hand hygiene, and seasonal influenza-like illness among young adults: a randomized intervention trial. J Infect Dis. 2010;201:491–8. PubMed https://doi.org/10.1086/650396

19. Aiello AE, Perez V, Coulborn RM, Davis BM, Uddin M, Monto AS. Facemasks, hand hygiene, and influenza among young adults: a randomized intervention trial. PLoS One. 2012;7:e29744. PubMed https://doi.org/10.1371/journal.pone.0029744

20. Zayas G, Chiang MC, Wong E, MacDonald F, Lange CF, Senthilselvan A, et al. Effectiveness of cough etiquette maneuvers in disrupting the chain of transmission of infectious respiratory diseases. BMC Public Health. 2013;13:811. PubMed https://doi.org/10.1186/1471-2458-13-811

21. Centers for Disease Control and Prevention. Respiratory hygiene/cough etiquette in healthcare settings. Influenza (flu), 2012 [cited 2019 Jul 10]. https://www.cdc.gov/flu/professionals/infectioncontrol/resphygiene.htm

22. World Health Organization. Infection-control measures for health care of patients with acute respiratory diseases in community settings: Trainer's guide, 2009 [cited 2019 Jul 10]. http://www.who.int/csr/resources/publications/ARDSTrainerweb.pdf

23. World Health Organization. Advice on the use of masks in the community setting in Influenza A (H1N1) outbreaks, 2009 [cited 2019 Jul 10]. http://www.who.int/csr/resources/publications/Adviceusemaskscommunityrevised.pdf

24. US Food and Drug Administration. Masks and N95 respirators, 2018 [cited 2019 Jul 10]. https://www.fda.gov/medicaldevices/productsandmedicalprocedures/generalhospitaldevicesandsupplies/personalprotectiveequipment/ucm055977.htm

25. Centers for Disease Control and Prevention. Respirator fact sheet, 2012 [cited 2019 Jul 10]. https://www.cdc.gov/niosh/npptl/topics/respirators/factsheets/respsars.html

26. Rengasamy S, Eimer BC, Shaffer RE. Comparison of nanoparticle filtration performance of NIOSH-approved and CE-marked particulate filtering facepiece respirators. Ann Occup Hyg. 2009;53:117–28. PubMed https://doi.org/10.1093/annhyg/men086

27. MacIntyre CR, Cauchemez S, Dwyer DE, Seale H, Cheung P, Browne G, et al. Face mask use and control of respiratory virus transmission in households. Emerg Infect Dis. 2009;15:233–41. PubMed https://doi.org/10.3201/eid1502.081166

28. MacIntyre CR, Zhang Y, Chughtai AA, Seale H, Zhang D, Chu Y, et al. Cluster randomised controlled trial to examine medical mask use as source control for people with respiratory illness. BMJ Open. 2016;6:e012330. PubMed https://doi.org/10.1136/bmjopen-2016-012330

29. Barasheed O, Almasri N, Badahdah AM, Heron L, Taylor J, McPhee K, et al.; Hajj Research Team. Pilot randomised controlled trial to test effectiveness of facemasks in preventing influenza-like illness transmission among Australian Hajj pilgrims in 2011. Infect Disord Drug Targets. 2014;14:110–6. PubMed https://doi.org/10.2174/1871526514666141021112855

30. Casas L, Espinosa A, Borràs-Santos A, Jacobs J, Krop E, Heederik D, et al. Domestic use of bleach and infections in children: a multicentre cross-sectional study. Occup Environ Med. 2015;72:602–4. PubMed https://doi.org/10.1136/oemed-2014-102701

31. Ibfelt T, Engelund EH, Schultz AC, Andersen LP. Effect of cleaning and disinfection of toys on infectious diseases and micro-organisms in daycare nurseries. J Hosp Infect. 2015;89:109–15. PubMed https://doi.org/10.1016/j.jhin.2014.10.007

32. Sandora TJ, Shih M-C, Goldmann DA. Reducing absenteeism from gastrointestinal and respiratory illness in elementary school students: a randomized, controlled trial of an infection-control intervention. Pediatrics. 2008;121:e1555–62. PubMed https://doi.org/10.1542/peds.2007-2597
